# Identification of prognostic biomarkers related to the tumor microenvironment in thyroid carcinoma

**DOI:** 10.1038/s41598-021-90538-3

**Published:** 2021-08-10

**Authors:** Jun-wei Du, Guo-quan Li, Yang-sen Li, Xin-guang Qiu

**Affiliations:** Department of Thyroid Surgery, The First Affiliated Hospital of Zhengzhou University, Zhengzhou, 450000 Henan China

**Keywords:** Computational biology and bioinformatics, Immunology

## Abstract

Thyroid Carcinoma (THCA) is the most common endocrine tumor that is mainly treated using surgery and radiotherapy. In addition, immunotherapy is a recently developed treatment option that has played an essential role in the management of several types of tumors. However, few reports exist on the use of immunotherapy to treat THCA. The study downloaded the miRNA, mRNA and lncRNA data for THCA patients from the TCGA database (https://portal.gdc.cancer.gov/). Thereafter, the tumor samples were divided into cold and hot tumors, based on the immune score of the tumor microenvironment. Moreover, the differentially expressed lncRNAs and miRNAs were obtained. Finally, the study jointly constructed a ceRNA network through differential analysis of the mRNA data for cold and hot tumors. The study first assessed the level of immune infiltration in the THCA tumor microenvironment then divided the samples into cold and hot tumors, based on the immune score. Additionally, a total of 568 up-regulated and 412 down-regulated DEGs were screened by analyzing the differences between hot and cold tumors. Thereafter, the study examined the differentially expressed genes for lncRNA and miRNA. The results revealed 629 differentially expressed genes related to lncRNA and 114 associated with miRNA. Finally, a ceRNA network of the differentially expressed genes was constructed. The results showed a five-miRNA hubnet, i.e., hsa-mir-204, hsa-mir-128, hsa-mir-214, hsa-mir-150 and hsa-mir-338. The present study identified the immune-related mRNA, lncRNA and miRNA in THCA then constructed a ceRNA network. These results are therefore important as they provide more insights on the immune mechanisms in THCA. The findings also provides additional information for possible THCA immunotherapy.

## Introduction

Thyroid Carcinoma (THCA) is a common endocrine malignant tumor whose incidence has been on the rise over the recent years. The disease is mainly divided into four types, namely; papillary carcinoma (85%), follicular carcinoma (10–15%), medullary carcinoma (5–10%) and undifferentiated THCA (< 5%)^1^. However, traditional treatment methods do not significantly improve the survival rate of patients, leading to the search for new treatment methods^2^. Notably, immunotherapy has been applied in many solid tumors, with satisfactory results although it has not be used extensively for the treatment of THCA. Therefore, it is important to explore the immune mechanisms in THCA in order to develop suitable immunotherapies.

Notably, Rudolf Virchow (the father of modern cytopathology) proposed the relationship between microinflammation and subsequent development of cancer, in 1863^3^. In addition, Paul Ehrlich in 1909 proposed the idea of using the immune system to control cancer^4^. The tumor microenvironment, especially the immune system, plays an essential role in regulating the progression of tumors and tumor response to treatment. It mainly stimulates tumor-specific immune responses by inducing the immunogenic death of tumor cells or participating in immune response mechanisms^5^. Moreover, tumors are divided into the cold and hot types based on the level of immune infiltration in the tumor microenvironment. The microenvironment of hot tumors is characterized by a higher degree of immune invasion and a more enhanced immune effect, with a strong antigen presentation ability and T cell activation. Such levels of immune infiltration lead to the production of tumor specific CD8 + T cells, which can clear cancer cells and generate systemic tumor specific immunity, resulting to a long-term anti-tumor memory response^6,7^.On the other hand, the microenvironment of cold tumors has no infiltration of immune cells or is mainly infiltrated by suppressive regulatory cell subtypes (including regulatory T cells (Tregs), regulatory B cells (bregs) and Myeloid Suppressor Cells (MDSCs))^8–10^. Consequently, the present study divided the tumor samples into two groups, namely; cold and hot tumors, based on the degree of immune invasion. The study further screened the differentially expressed genes for lncRNA, miRNA and mRNA between the two groups in order to assess the immune mechanisms related to THCA.

The Cancer Genome Atlas (TCGA) database can be used for the large-scale analysis of global gene expression profiles and database mining, to assess the potential correlation between genes and the overall survival rate of various malignant tumors^11^. In this study, the miRNA, mRNA and lncRNA data for THCA patients was downloaded from the TCGA database. The study also downloaded the clinical information corresponding to the miRNA data for the THCA patients. In addition, the ssGSEA, MCP counter, CIBERSORT and X-cell packages were used to evaluate the immune cells in the THCA tumor samples and normal samples. The Xcell algorithm and estimate package were also used to evaluate the immune and stromal scores. Furthermore, the THCA samples were divided into four subtypes through congruent clustering, in order to understand the differences in immune cell types, immune-related molecules, tumor size distribution and grading, in the four subtypes. Based on differences in the immune and stromal scores, Clusters 3 and 4 were defined as hot tumors while and Clusters 1 and 2 were considered to be cold tumors. Thereafter, differential analysis of cold and hot tumors, enrichment analysis of the differentially expressed genes and construction of the protein interaction network was conducted.

Furthermore, the competitive endogenous RNA (ceRNA) includes protein coding RNA, tRNA, rRNA, long non coding RNA (lncRNA), pseudogene RNA and circular RNA^12^. Therefore, the study compared the expression of lncRNA and miRNA in cold and hot tumors. Finally, the differentially expressed lncRNA, miRNA and mRNA obtained were used to construct the immune-related ceRNA network. The study identified five immune connected ceRNA networks in THCA, which are important in understanding the mechanism of immune invasion and immunotherapy.

The present study aimed to construct a ceRNA regulatory network using microarray data collected from a public database and preliminarily identify the regulatory mechanism mediated by a novel lncRNA, miRNA and mRNA in THCA. The study therefore highlights possible targets for the development of new therapeutic strategies against THCA.

## Methods

### Data download and assessment of immune infiltration

The miRNA, mRNA and lncRNA data for THCA patients was downloaded from the TCGA official website (https://portal.gdc.cancer.gov/). The data included 57 normal and 511 tumor samples. Notably, there are four commonly used methods to evaluate immune cell infiltration in the tumor microenvironment, namely: single sample Gene Set Enrichment Analysis (ssGSEA), the Microenvironment Cell Populations (MCP)-counter, CIBERSORT and Xcell^13–16^. However, the study used all the four methods in order to minimize errors. The inclusion criteria included; *p* < 0.05 (*p* value < 0.1 was used in CIBERSORT to obtain enough samples). Moreover, the “ggplot2” package was used for plotting.

### Evaluation of immune and stromal scores in the tumor and paracancerous samples

The Xcell method was used to calculate the immune and stromal scores in the tumor microenvironment. It is noteworthy that immune and stromal cells are two major types of non-tumor components that have been proposed to be valuable for the diagnosis and diagnostic assessment of tumors. Inclusion criteria: *p* < 0.05. The “ggplot2” package was used to draw violin diagrams to visualize the differences in infiltration of immune cell.

### Correlation of immune cells

Results from the four evaluation methods were used to calculate the correlation between different immune cells. Therefore, blue was used to represent positive correlation while red represented negative correlation. Correlation analysis of the immune cells in THCA showed that the interaction between adjacent and cancer cells was significantly different.

### Cell consistent clustering

The cell consistent clustering method was used to divide the THCA samples into different subtypes, based on the level of immune infiltration. In addition, the Cumulative Distribution Function (CDF) was employed to identify the optimal number of subsets. Finally, four different subtypes were identified and a heat map was used to compare the immune stromal score and distribution of immune cells in the different subtypes. Classification of osteosarcoma patients in to various clinically significant subtypes was performed using the "ConsensusClusterPlus" package (http://www.bioconductor.org/). The clusters were visualized using a heat map and dela diagram.

### Differences in the immune stromal score, immune-related molecules, tumor size distribution and grading in the different subtypes

The progression and metastasis of tumors depend on the two-way interaction between cancer cells and their environment, forming the Tumor Microenvironment (TME)^17^. Notably, the TME is usually different in different stages of tumor progression and this can either promote or inhibit the formation of tumors. It is also known that immune cells can be activated to promote the formation and progression of tumors^18^. Additionally, the immune stromal score can predict the level of immune invasion in the tumor microenvironment. Therefore, the present study compared the immune stromal score, expression of immune-related molecules, tumor size distribution and grade in the four subtypes of immune cell infiltration.

### Analysis of the difference between cold and hot tumors

The progression of cancer requires tumor cells to be immune tolerant. Therefore, tumors can be divided into two subtypes according to the infiltration of T lymphocytes^19,20^. In addition, different proportions of effector T cells and regulatory T cells in thermal tumors reflect different degrees of immunosuppression, which affects the progression of tumors^21^. The study compared the immune stromal scores among the different subtypes. Consequently, Clusters 3 and 4 which had higher immune stromal scores were classified as hot tumors while Clusters 1 and 2 were considered to be cold tumors. Moreover, the Differentially Expressed Genes (DEGs) between the controls and patients with DTC were generated using the LIMMA method^22^, where statistical significance was set at |log-fold change (logFC)|> 1 and Benjamini and Hochberg-corrected False Discovery Rates (FDR) < 0.05. Furthermore, a hierarchical cluster heatmap based on the Euclidean distance was generated using the pheatmap package in R (Version: 1.0.12) (R Core Team. (2019). R: A language and environment for statistical computing. R Foundation for Statistical Computing, Vienna, Austria: URL http://www.R-project.org/). The Euclidean distance represented the expression intensity and direction of DEGs.

### Enrichment analysis of differentially expressed genes and construction of a protein interaction network

The Gene Ontology (GO) and Kyoto Encyclopedia of Genes and Genomes (KEGG)^23–25^pathway enrichment analyses were performed using the clusterprofiler package^26^. The selection criteria included; logFC > 1 or < −0.5 and adjusted *p* value s < 0.05. Additionally, the proteins encoded by the DEGs and data for the PPI network were obtained using the Search Tool for the Retrieval of Interacting Genes (STRING) database (http://string-db.org). Moreover, the Cytoscape software (version 3.7.0; http://cytoscape.org/) was used to visualize the interactions among the candidate DEGs^27^.

### Search for tumor related regulatory molecules

The differentially expressed genes between tumor and adjacent tumor tissues were obtained. Thereafter, the genes up-regulated in cold tumors (adjusted *p* value < 0.05) were crossed with those overexpressed in cancer. These genes were related to cancer and played a role in negative immune regulation. Similarly, the up-regulated genes in hot tumors were crossed with those expressed in low levels in cancer, which are positive immune regulatory genes. Finally, GO and KEGG enrichment analyses were used to obtain the immune regulatory molecules.

### Differences between lncRNA and miRNA in cold and hot tumors

Differential analysis (up: | log2fc |> 1, down: | log2fc |> 1, adjusted *p* value < 0.05) was performed by comparing the cold and hot tumors using the limma package in R.

### Construction of hub ceRNA network

The study constructed a co-expression network for DElncRNAs, DEmiRNAs and DEmRNAs in order to assess the functions of the lncRNAs, miRNAs and mRNAs in the ceRNA network and to further improve the reliability of the network. Thereafter, the ggalluvial package in R (Version: 0.9.1) was used to visualize the ceRNA network^28^. Notably, miRNAs are 19-23nt short RNAs transcribed from endogenous transcriptomes and distributed throughout the cell^29^. On the other hand, long non coding RNAs (lncRNAs) are involved in a variety of cellular functions, most of which require interaction with one or more RNA Binding Proteins (RBPs)^30^. Herein, DEmRNAs targeted by the DEmiRNAs were retrieved from the miRDB (Version 5.0; http://mirdb.org), miRTarBase (Version 7.0; http://mirtarbase.mbc.nctu.edu.tw/), and TargetScan (Version 7.2; http://www.targetscan.org/vert_72/) databases^31–33^. Moreover, the Cytoscape software (https://cytoscape.org/) was used to visualize the relationships in the ceRNA network. Additionally, the target genes for miRNA were predicted using mirdb, targetscan and mirtarbase. The study also constructed an immune-related ceRNA network with different lncRNA, miRNA and mRNA then defined the first five networks as the hubnet, using the cytohub module. The regulatory relationship between transcription factors and miRNA has a significant effect on genes, and the transcription factors that regulate hub miRNA can be predicted from GeneCards(https://www.genecards.org/).

### Prediction of PD1/PDL-1 related immune cells and hub prognostic genes

As we all know, the immune checkpoint PD1 and its ligand PDL1 combine to help tumor cells avoid immune killing. In thyroid cancer, we first assessed the relationship between Programmed cell death protein 1 (PDCD1) and CD274 Molecule (CD274) and patient prognosis. In addition, in order to explore the relationship between PD1/PDL-1 and immunity in thyroid cancer, the correlation between PD1/PDL-1 and immune cells were calculated using the “ssGSEA”, “MCP-counter”, “CIBERSORT” and “Xcell” four packages in R software. Finally, in order to further screen the possible prognostic markers of THCA, we analyzed the survival of all genes in thyroid cancer and selected the first five survival-related hub genes based on the log-rank *p* value. “Survival” and “survminer” packages are used for survival analysis.

### Ethical statement

The present study obtained data from the TCGA and did not include any animal experiment or human specimens. Ethical approval was therefore not required.

## Results

### Data download, evaluation of immune invasion and comparison of immune cells between cancer and adjacent tissues

**Figure 1:**
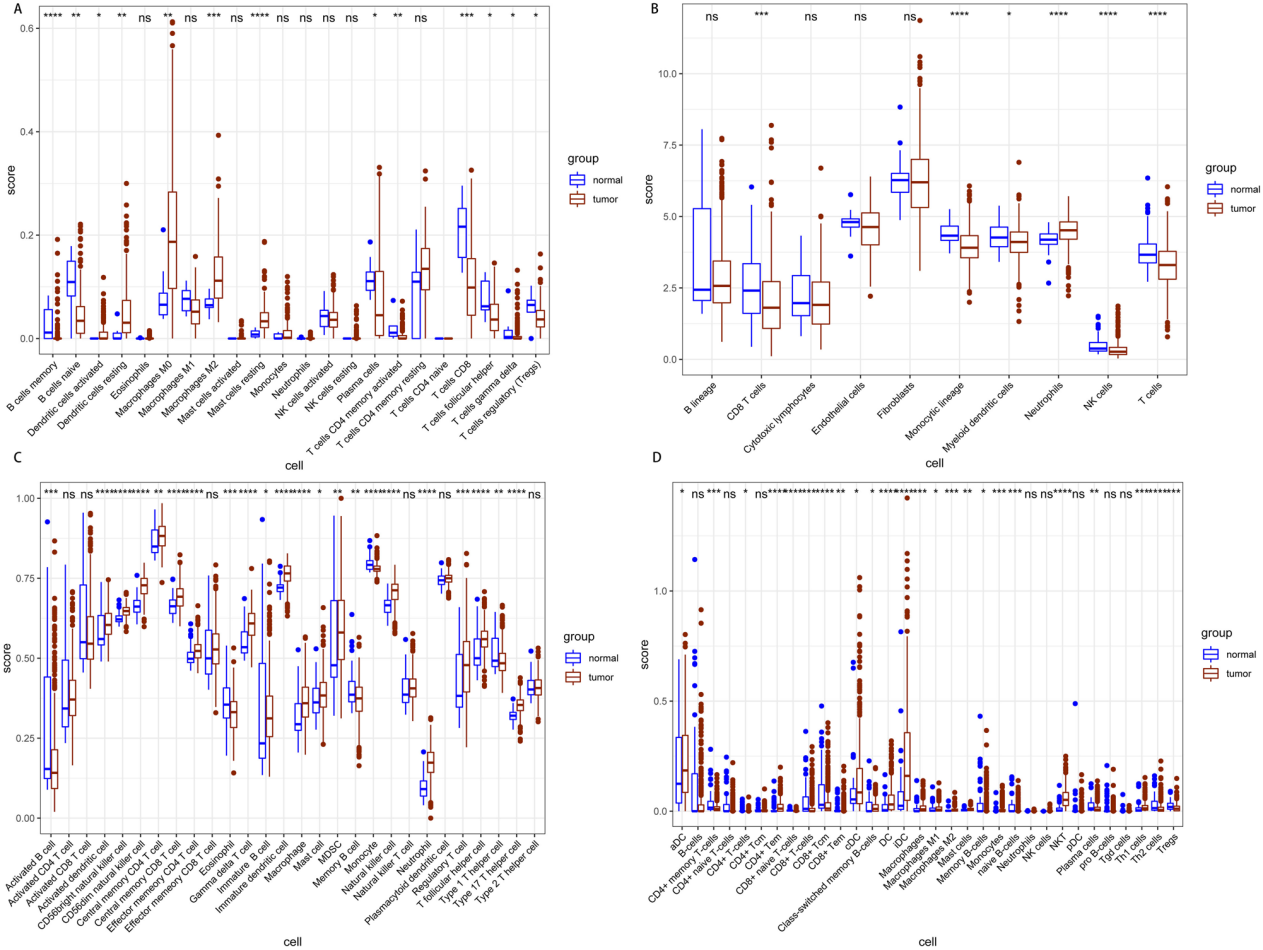
Four methods assessed the number of immune cells in cancer and adjacent tissues. (**A**) The CIBERSORT evaluation method showed that the number of B cells and T-cells in cancer tissues was less than that in the adjacent tissues. However, cancer tissues had a higher number of dendritic and mast cells than that the adjacent tissues. (**B**) The MCP-counter evaluation method showed that cancer tissues had a lower number of CD8 T cells, monocytes, myeloid dendritic cells, NK cells and T cells than that the adjacent tissues. However, the number of neutrophils was higher in cancer tissues. (**C**) The ssGSEA evaluation method showed that the number of most T cells and B cells in cancer tissues was lower than that in adjacent tissues. However, the cancer tissues had a higher number mast cells, monocytes and neutrophils. (**D**) The Xcell evaluation method showed that the number of B cells, T-cells and monocytes was lower in cancer tissues than that in the adjacent tissues. However, cancer tissues had a higher number of dendritic cells, mast cells and natural killer cells.

The study downloaded the miRNA, mRNA and lncRNA data for THCA patients from the TCGA database. In addition, four methods, namely; ssGSEA, MCP-counter, CIBERSORT and Xcell, were used to compare the levels of immune cells between cancer and adjacent tissues. The four methods revealed different numbers of immune cells (CIBERSORT: 22, ssGSEA: 28, MCP counter: 10, Xcell: 67). In order to reduce possible errors, the study used all the four evaluation methods. The results showed that cancer tissues had significantly more numbers of immune cells than the paracancerous ones (Fig. 1A–D).

### Correlation of immune cells

**Figure 2:**
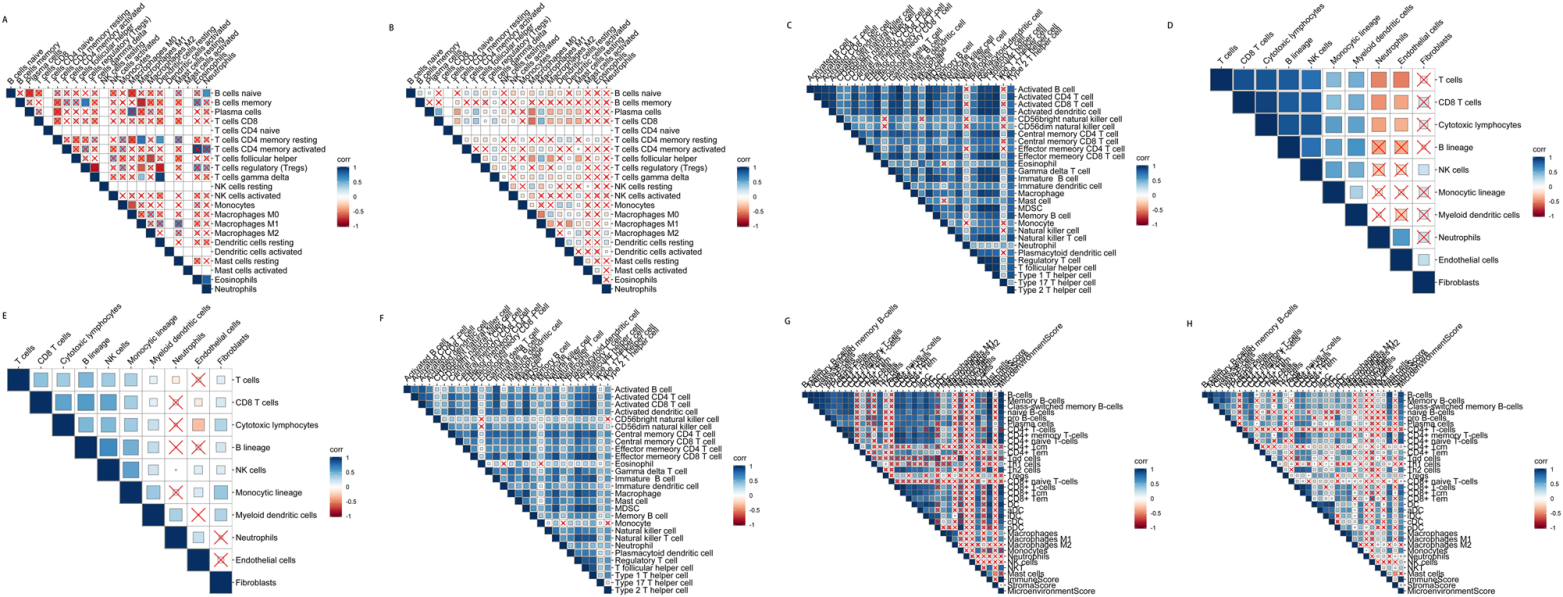
Correlation between immune cells. The correlation between immune cells was assessed using four different methods and is shown in (**A**–**H**). Blue represents positive correlation and red depicts negative correlation.

The study analyzed the correlation between immune cells in tumor and paracancerous samples (Fig. 2A–H). The results showed that the correlations between immune cells in tumor and paracancerous samples were significantly different, possibly due to the synergistic infiltration of immune cells activated by cancer antigens. In tumor tissues, the synergistic effect of different immune cells constitutes the tumor immune microenvironment and plays an important role in the invasion and development of tumors.

### Cell consensus clustering

**Figure 3:**
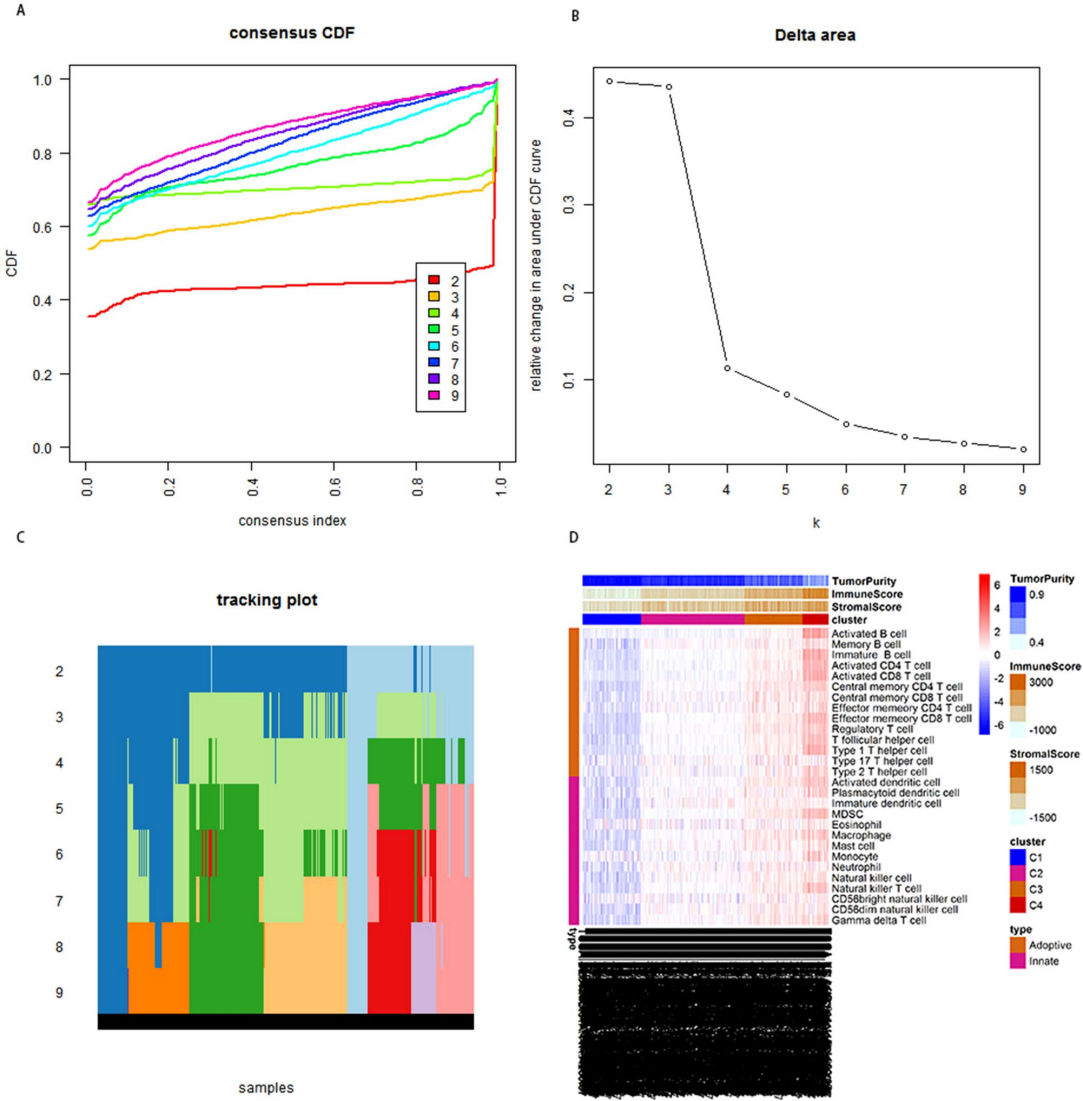
The consistent cluster map of immune cells. CDF (cumulative distribution function) showed that a k value of 4 was the optimal number for the subgroups (**B**). In addition, a heat map was drawn to show differences in tumor purity, immune and stromal scores among the different subtypes (**D**).

The THCA samples were classified into different subtypes based to the immune infiltration levels. In addition, the Cumulative Distribution Function (CDF) was used to identify the number of optimal subgroups (Fig. 3A–C). Finally, four different subtypes were identified. Additionally, the distribution of cells and immune stromal scores were compared between different subtypes using a heat map. The study also assessed the differences in tumor purity, immune and stromal scores in innate and adaptive immune mechanisms, among the four subtypes. The results showed that Clusters 3 and 4 had lower tumor purity than the other two subtypes. However, Clusters 3 ad 4 had higher immune and stromal scores than the other two subtypes (Fig. 3D).

### Immune scores and immune-related molecules in different subtypes

**Figure 4:**
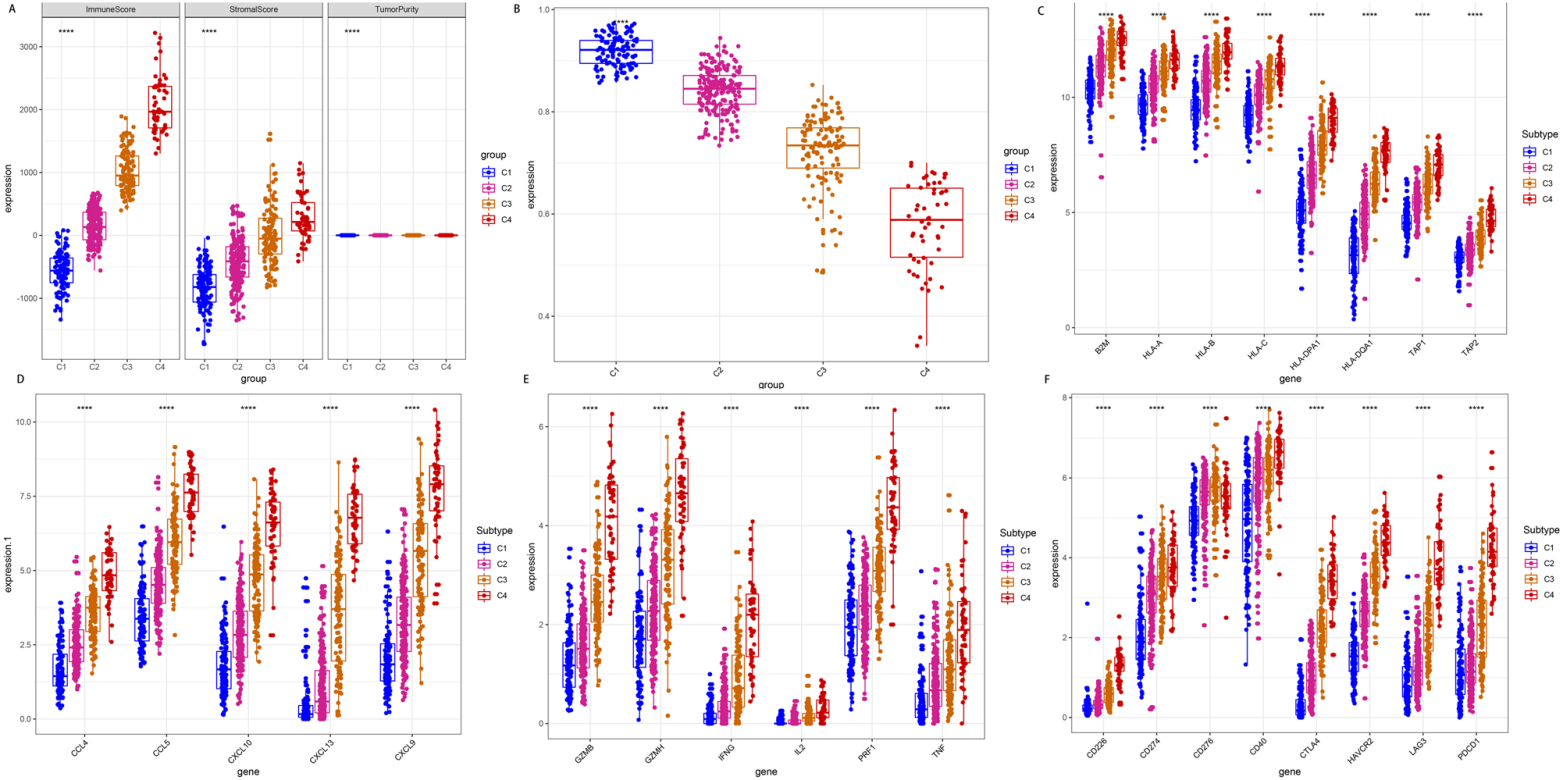
A box plot of immune scores and expression levels of immune-related molecules in the different subtypes. (**A**,**B**) The immune scores and immune matrix scores in the four clusters. Clusters 3 and 4 had higher immune scores than Clusters 1 and 2. (**C**–**F**) The expression levels of multiple immune molecules were compared in the four clusters. The expression levels of the molecules were higher in Clusters 3 and 4. *, *p* < 0.05. **, *p* < 0.01. ***, *p* < 0.001. ****, *p* < 0.0001.

The immune score can be used to assess the level of immune infiltration in the tumor microenvironment. Therefore, the study compared the immune stromal scores and the expression levels of immune-related molecules in 4 different immune infiltrating subtypes (Fig. 4A–F). Based on the ESTIMATE algorithm, the stromal score ranged from − 600 to 1700 while the immune score was between -800 and 3500. Additionally, Clusters 3 and 4 had significantly higher immune scores than Clusters 1 and 2. The results therefore suggested that immune and stromal scores are important in the classification of subtypes.

### Differences in tumor size distribution and grade

**Figure 5:**
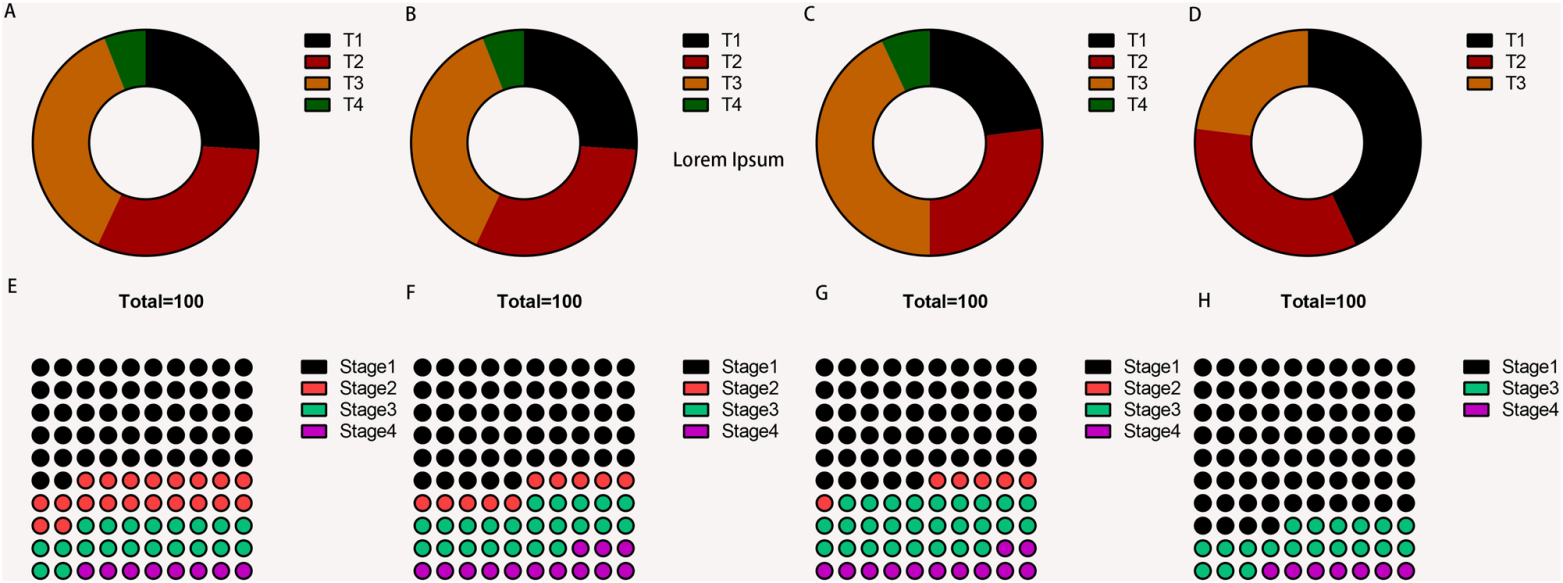
Tumor size distribution and grading in different subtypes. (**A**–**H**) The distribution ratio and grading of thyroid tumor sizes in different clusters. The ratio of Cluster 4 in T1 and Stage 1 was significantly higher than that of the other clusters.

The American Joint Committee on Cancer/International Cancer Control (AJCC/UICC) recently released the 8th edition of the TNM staging system^34^. Notably, T represents the size of tumor, N represents the level of lymph node metastasis and M shows the presence or absence of distant metastasis. However, the TNM staging system alone is not enough to accurately reflect the stage of tumors. Therefore, other tumor-related factors have been explored, leading to the identification of different grades of tumors (Stages 1–4)^35^. The present study used a pie chart to understand the differences in tumor size distribution and grading between the different THCA subtypes (Fig. 5A–H). The findings showed that the ratio of Cluster 4 at T1 and Stage1 was significantly higher than that of the other clusters, suggesting that hot tumors have lower pathological stages than cold tumors.

### Analysis of differences between cold and hot tumors

**Figure 6:**
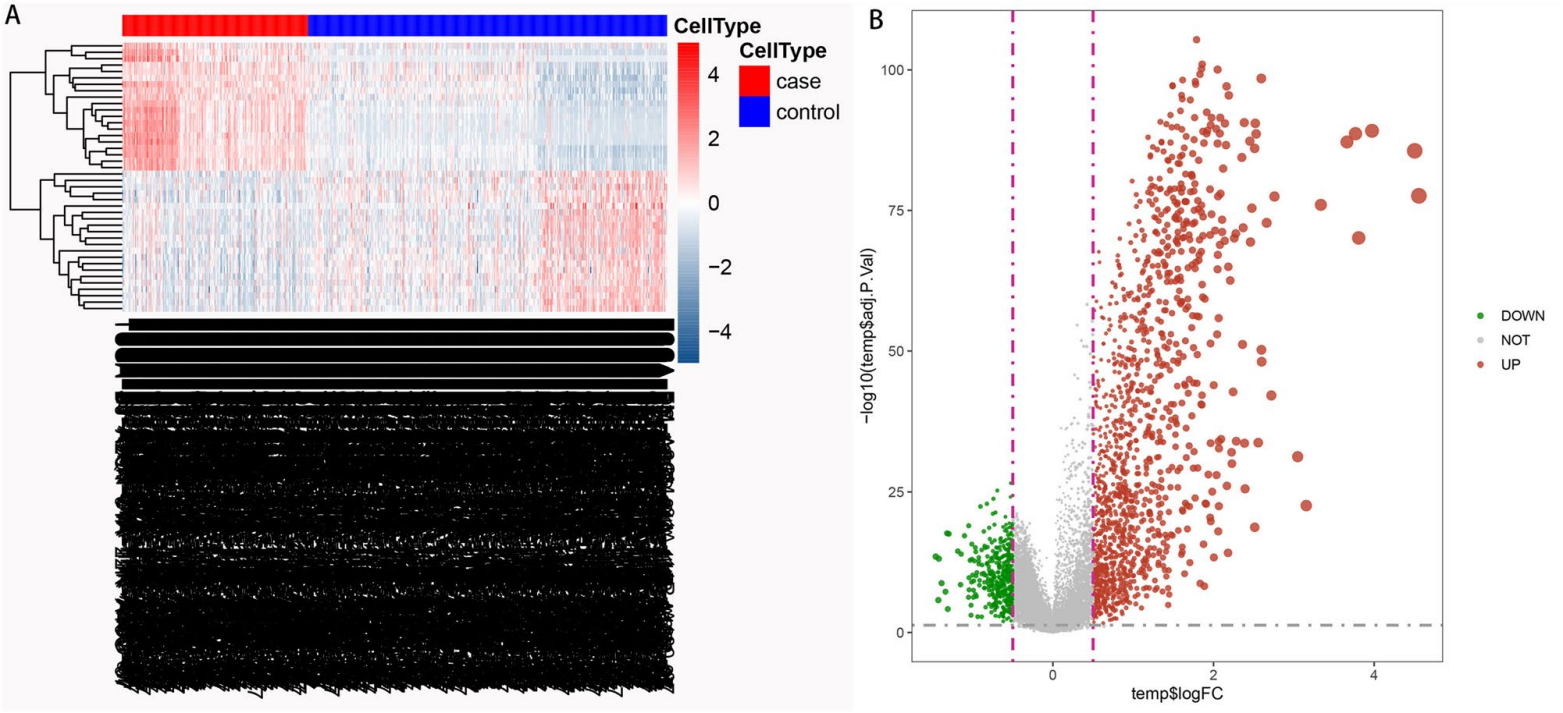
Differences in immune cell types and differentially expressed genes in cold and hot tumors. (**A**) The different immune cell types in cancer and adjacent tissue samples. (**B**) The up-regulation and down-regulation of differentially expressed genes in cold and hot tumors.

Tumors in Clusters 3 and 4 were defined as hot tumors because they had higher immune stromal scores while those in Clusters 1 and 2 were considered to be cold tumors because they had lower immune stromal scores. Additionally, the heat map showed that the immune cell types of THCA and those of adjacent samples were significantly different (Fig. 6A). The study also used the limma package (|Log2FC|> 2, adjusted *p* value  < 0.05) to identify differentially expressed genes then drew a volcano map to examine the differences in gene expression, between cold and hot tumors (Fig. 6B).

### Enrichment analysis of DEGs and construction of protein interaction network for the DEGs

**Figure 7:**
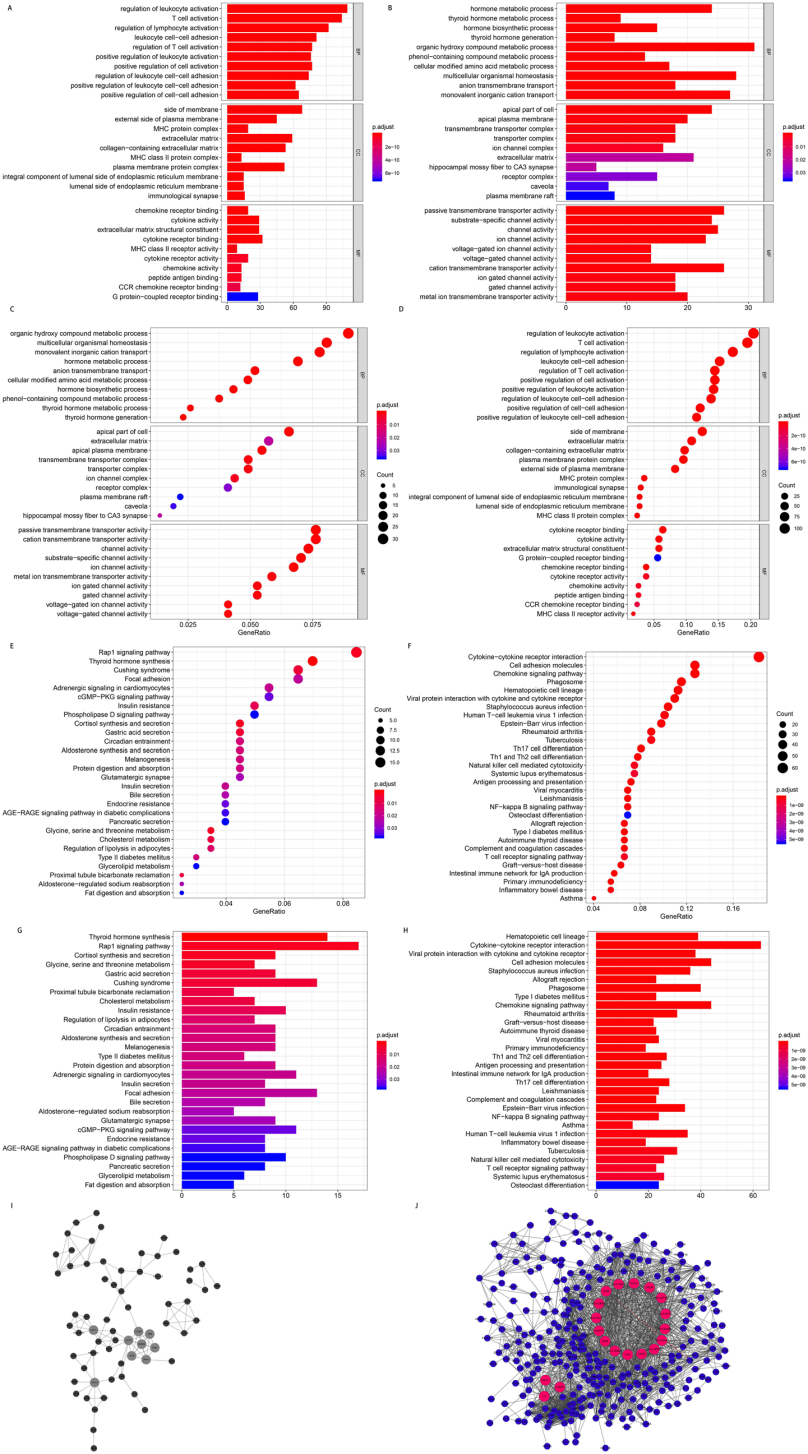
The GO, KEGG and PPI analysis of DEGs related to cold and hot tumors. (**A**–**D**) GO analysis of DEGs related to cold and hot tumors. (**E**–**H**) KEGG pathway analysis of DEGs related to cold and hot tumors. (**I**,**J**) PPI analysis of DEGs related to cold and hot tumors. (**I**) The down-regulated genes. (**J**) The up-regulated genes. BP, Biological Process; CC, Cellular Component; MF, Molecular Function; GO, Gene Ontology; KEGG, Kyoto Encyclopedia of Genes and Genomes; PPI, Protein–protein Interaction; DEGs, Differentially Expressed Genes.

The results revealed 568 up-regulated and 412 down-regulated genes. Thereafter, the clusterprofiler package was used to conduct GO enrichment and KEGG pathway enrichment analyses of the differentially expressed genes related to cold and hot tumors^36^. The findings showed that the up-regulated genes were mainly enriched in the regulation of leukocyte activation (GO: 0002694), T cell activation (GO: 0042110), regulation of lymphocyte activation (GO: 0051249) and leukocyte cell–cell adhesion (GO: 0007159). On the other hand, the down-regulated genes were mainly enriched in the hormone metabolic process (GO: 0042445), thyroid hormone metabolic process (GO: 0042403), hormone biosynthetic process (GO: 0042446) and thyroid hormone generation (GO: 0006590), as shown in Fig. 7A–D. Additionally, KEGG pathway enrichment analysis showed that the up-regulated differential genes were mainly enriched in the hematopoietic cell lineage (hsa04640), Cytokine-cytokine receptor interaction (hsa04060) and viral protein interaction with cytokine and cytokine receptor (hsa04061). On the other hand, the down-regulated differential genes were mainly enriched in thyroid hormone synthesis (hsa04918), the Rap1 signaling pathway (hsa04015) and Cortisol synthesis and secretion (hsa04927), as shown in Fig. 7E–H. Furthermore, the STRING online tool was used to construct a Protein–protein Interaction (PPI) network of the differentially expressed genes related to cold and hot tumors. Notably, the up-regulated differential genes with a node connection greater than 20 were considered to be hub genes. On the other hand, the down-regulated differential genes with a node connection greater than 10 were defined as hub genes (Fig. 7I: the protein interaction network of the down-regulated differential genes; Fig. 7J: the protein interaction network of the up-regulated differential genes). The analysis revealed 19 up-regulated and nine down-regulated hub genes. Moreover, the protein interaction network revealed a wide range of links between the markers related to the stromal scores and the markers related to immune scores in the differentially expressed genes, which may be related to the higher immune and stromal scores in hot tumors.

### The tumor-related regulatory molecules and enrichment analysis of tumor-related molecules

T cell-based cancer immunotherapy, including checkpoint suppression or adoptive cell therapy, has greatly revolutionized cancer treatment^37^. It is also well known that immunity plays a significant role in the occurrence and development of tumors. The present study first assessed the genes differently expressed in cancer and the adjacent tissues then crossed the genes up-regulated in cold tumors (adjusted *p* value  < 0.05) with those highly expressed in cancer. A total of 717 genes were identified to be negatively regulated immune genes, related to cancer. Therefore, these genes may be therapeutic targets.

**Figure 8:**
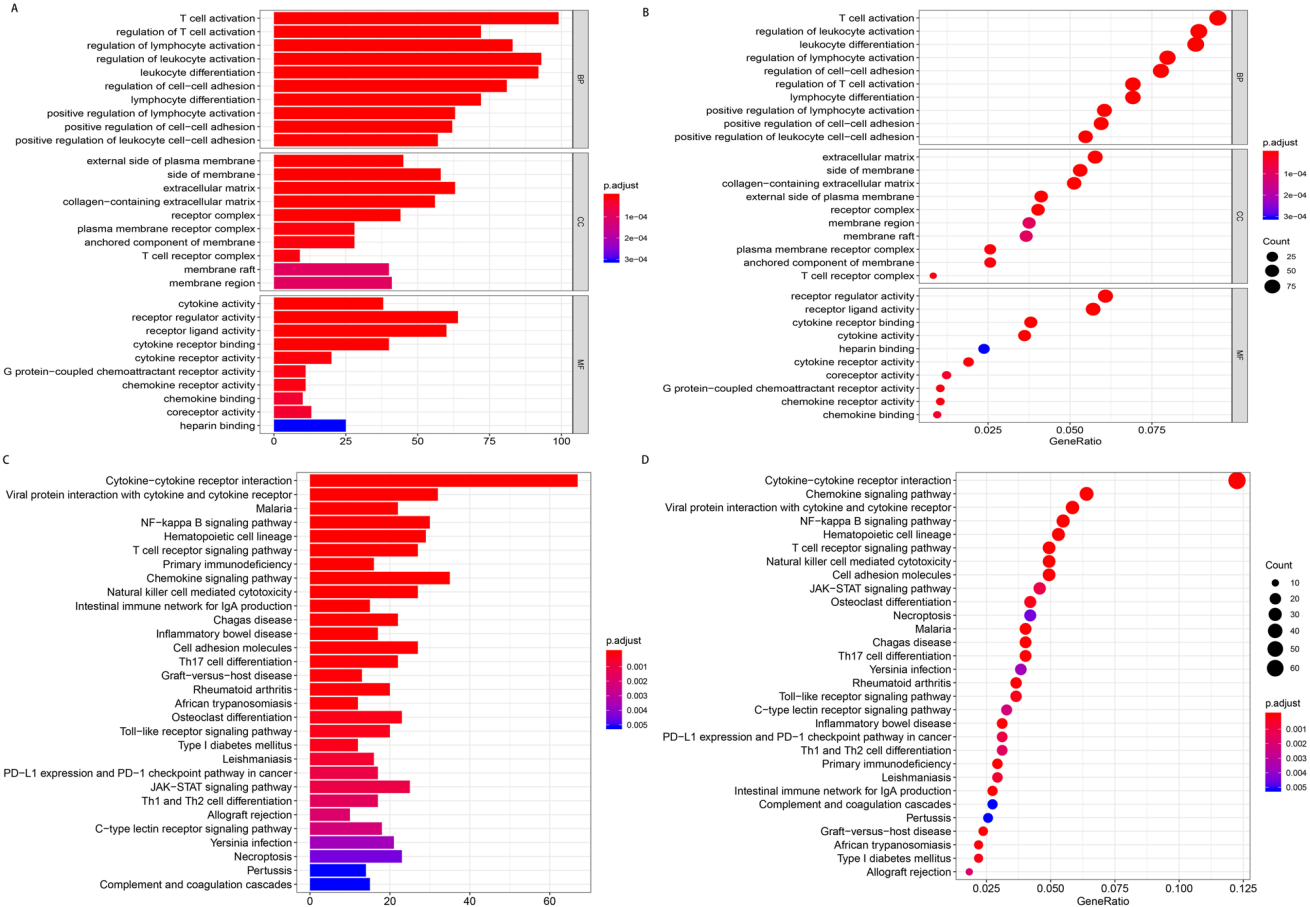
GO and KEGG enrichment analysis of immune-related genes. (**A**,**B**) The up-regulated DEGs were functionally enriched in GO. (**C**,**D**) The main pathways of the up-regulated DEGs were enriched in KEGG.

Similarly, the genes that were up-regulated in hot tumors (adjpvalue < 0.05) were crossed with those expressed in low levels in cancer and a total of 1246 genes were identified. These genes are positively regulated immunity, so agonists need to be added during the treatment. In addition, the results of GO enrichment analysis showed that the up-regulated genes were mainly enriched in T cell activation (GO:0,042,110), regulation of T cell activation (GO: 0050863), regulation of lymphocyte activation (GO: 0051249), external side of the plasma membrane (GO: 0009897) and cytokine activity (GO: 0005125), as shown in Fig. 8A,B. On the other hand, KEGG pathway enrichment analysis showed that the up-regulated genes were mainly enriched in Cytokine-cytokine receptor interaction (hsa04060) and viral protein interaction with cytokine and cytokine receptor (hsa04061), as shown in Fig. 8C,D. The down-regulated differential genes were however not enriched in related pathways.

### Differences between lncRNA and miRNA in cold and hot tumors

**Figure 9:**
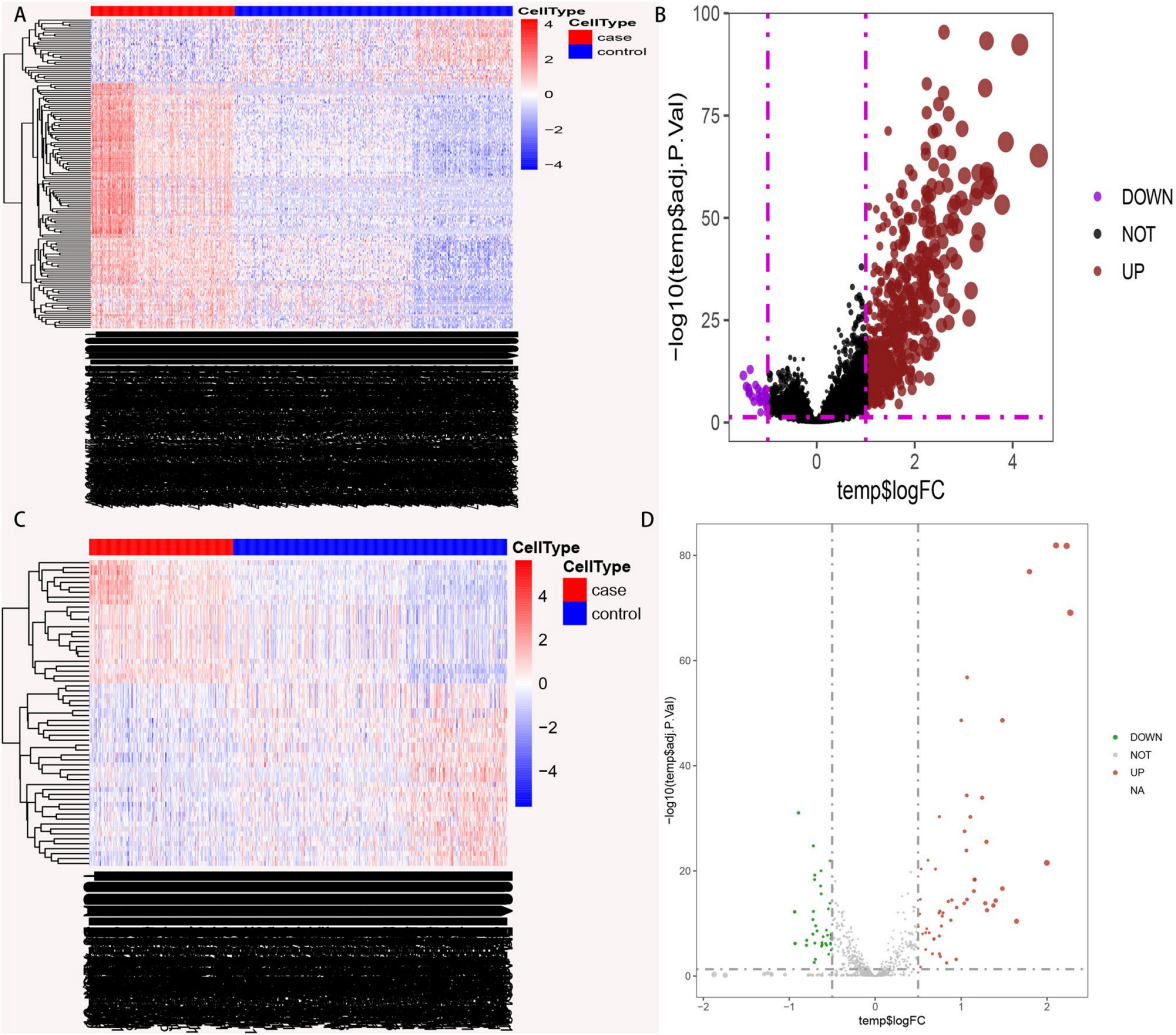
Differences in the expression of lncRNA and miRNA in cold and hot tumors. (**A**,**B**) Differential expression of lnRNA in cold and hot tumors (|Log2FC|> 1, adjusted *p* < 0.05). (**C**,**D**) Differential expression of miRNA in cold and hot tumors(|Log2FC|> 0.5, adjusted *p* value  < 0.05).

The study then compared the expression lncRNA and miRNA between the cold and hot tumors (Fig. 9A–D). In addition, the limma package was used to identify the differentially expressed genes. Thereafter, volcano maps were drawn to compare the differentially expressed genes between cold and hot tumors. The results revealed a total of 629 differential lncRNAs (|Log2FC|> 1, adjusted *p* < 0.05) and 114 differential miRNAs (|Log2FC|> 0.5, adjusted *p* value  < 0.05).

**Figure 10:**
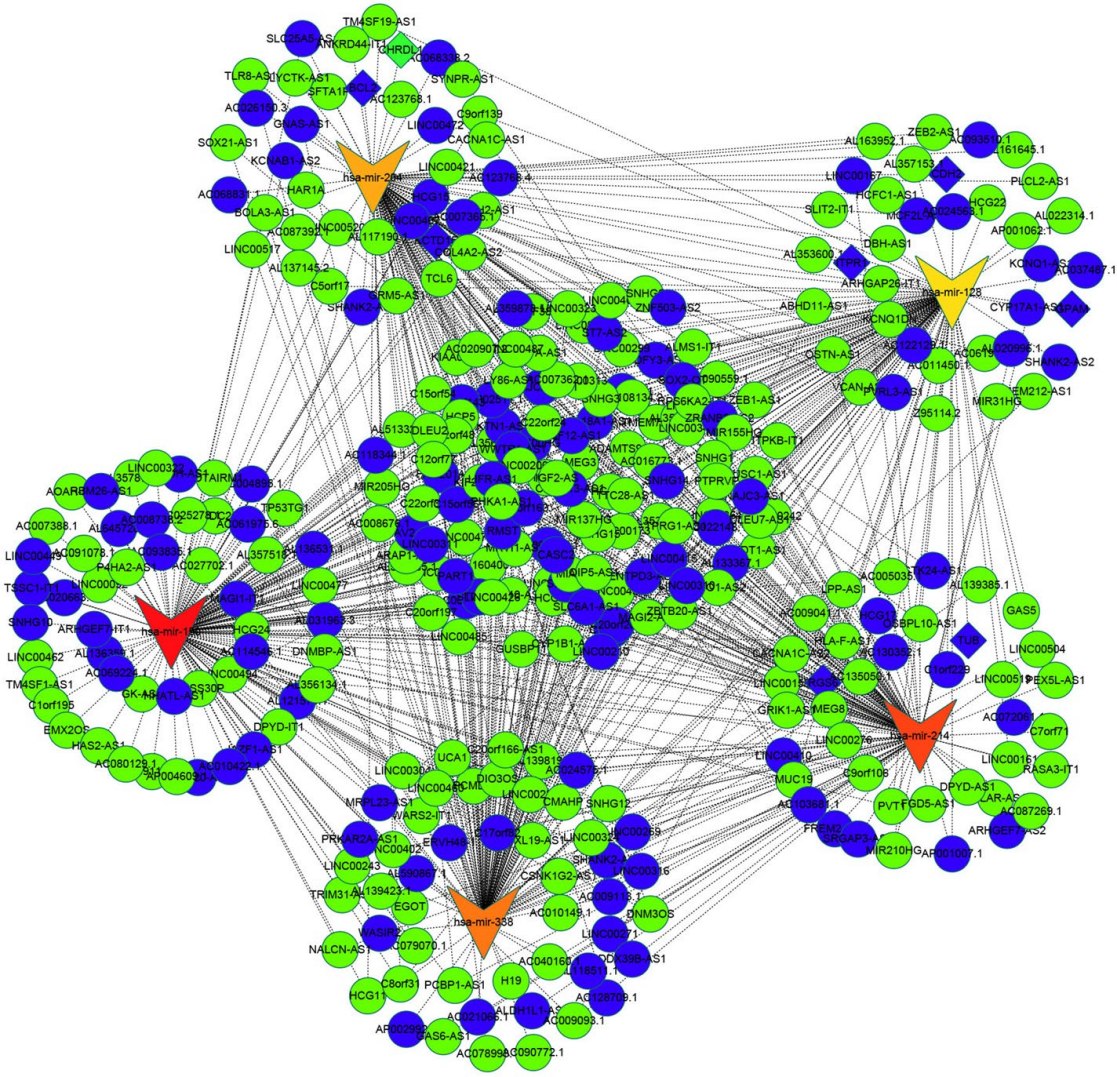
Construction of a ceRNA network. A ceRNA network consisting of 629 lncRNAs, 114 miRNAs and 980 mRNAs was constructed. The first five networks were then selected and defined as the hubnet. The circle represents lncRNA (adjusted < 0.05), the diamond represents mRNA (adjusted *p* < 0.05, logFC > 1, logFC < 0.05) and the triangle depicts miRNA (adjusted *p* < 0.05).

In order to understand the association of lncRNAs, mRNAs and miRNAs in DTC, the study built a ceRNA network based on the data mentioned above then used the ggalluvial package in R (Version: 0.9.1) to visualize the network. In addition, the miRNA target genes were assessed using the miRDB, Targetscan and miRTarBase databases. During the analysis, a circle represented lncRNA (adj < 0.05), a diamond represented mRNA (adj < 0.05, logFC > 1, logFC < 0.05) and a triangle was used to depict miRNA (adjusted *p* < 0.05). Finally, the cytohub module was used to calculate the first five networks which were defined as the hubnet (Fig. 10). Notably, five miRNAs, namely; hsa-mir-204, hsa-mir-128, hsa-mir-214, hsa-mir-150 and hsa-mir-338 were located in the central area of the network and were of great significance in THCA immunity. (Supplementary Table 1) We predicted the transcription factors of hsa-mir-204, hsa-mir-128, hsa-mir-214, hsa-mir-150 and hsa-mir-338 in GeneCards.

### Prediction of PD1/PDL-1 related immune cells and hub prognostic genes

We first assessed the prognostic relationship between PDCD1 and CD274 in patients with thyroid cancer. We found that high PDCD1 expression may be associated with better survival trend (*p* = 0.056) (Supplementary Fig. 1). Then we explored the correlation between PDCD1 and CD274 and immune cells in thyroid cancer. In Supplementary Fig. 2, the results of the four algorithms ssGSEA, MCP-counter, CIBERSORT and Xcell showed that PDCD1 and CD274 are significantly related to a variety of immune cells (Activated CD4 T cells, Activated CD8 T cells, Activated dendritic cells and NK cells). Finally, to find prognostic makers of THCA, we performed survival correlation analysis on all genes in samples of thyroid cancer patients, and finally determined five hub genes based on the log-rank value: CD47, CILP, DERA, KLHL33, PSMB8 (Supplementary Figs. 3–7).

## Discussion

Thyroid Carcinoma is one of the most common malignant tumors. In addition, screening of RNA transcripts has been conducted over the past 20 years and lncRNAs as well as miRNAs were shown to be strongly associated with tumorigenesis and metastasis in THCA^38–40^. Interactions between tumor cells and various components of the TME are significant and contribute to all the hallmarks of cancer^41^. Additionally, the TME can affect the growth and spread of tumors. Therefore, identifying the critical genes in the THCA microenvironment is important for the appropriate management and treatment of the cancer. Moreover, analysis of immune infiltration in the TME is significant for immune-related treatment of THCA. Consequently, the present study aimed to identify immune-related mRNAs, lncRNAs and miRNAs and further explore the relationship between these RNAs.

Existing evidence suggests that lncRNAs play a vital role in biological functions through multiple levels of regulation, including transcriptional, post-transcriptional, and epigenetic regulation^42,43^. Numerous studies have also shown that there is a complex and closely related regulatory network between miRNA and lncRNA. For instance, the relationship between miRNA and lncRNA was established in triple negative breast cancer^44^. Moreover, the ceRNA hypothesis was proposed to explain the mechanism of tumorigenesis. The hypothesis suggests that lncRNAs with sequences similar to their target miRNA can regulate mRNA expression by acting as a sponge of miRNA. This hypothesis therefore provides a novel theoretical insights and suggests valuable strategies as well as research directions for the diagnosis and treatment of malignancies^45^.

There are currently few studies on ceRNA networks related to THCA. Therefore, the present study downloaded mRNA, lncRNA and miRNA data of patients with THCA from the TCGA. The samples were then divided into the cold and hot tumors, through immune evaluation^46^. The microenvironment of hot tumors usually has a high level of immune infiltration and the immune effect is often relatively higher, with a strong antigen presentation ability and T cell activation. Such levels of immune infiltration in turn lead to the production of tumor-specific CD8 + T cells, which can eliminate cancer cells and generate systemic tumor-specific immunity, forming a long-term anti-tumor memory response^6,7^. However, "cold" tumors have no immune cell infiltration in the tumor microenvironment or are mainly infiltrated by inhibitory regulatory cell subtypes (including regulatory T cells (Tregs), regulatory B cells (Bregs) and MDSCs)^8–10^. This in turn results to the inhibition of cancer growth. Moreover, the study used DEGs to construct a ceRNA network then identified a hubnet consisitng of five miRNAs, namely; hsa-mir-204, hsa-mir-128, hsa-mir-214, hsa-mir-150 and hsa-mir-338. The complex relationship between transcription factors and miRNAs' regulation of genes is worthy of further exploration. We have predicted five hub miRNAs-related regulatory transcription factors (hsa-mir-204: ZNF341/JUND/SCRT1/TRIM28/EZH2, hsa-mir-128: SP1/BCL11A/IRF4/EBF1/CBFB, hsa-mir-214: CEBPB/MAX/EP300/GABPA/STAT3, hsa-mir-150: CHD2/ZBTB10/SP1/MXD4/FEZF1, hsa-mir-338: USF2/KLF1/HIC1). Notably, miRNAs are RNA molecules with a length of approximately 22 nucleotides. They bind to the 3'-untranslated region (3'-UTR) of their respective target genes and exert their effect on gene expression by inhibiting protein translation degrading mRNA^47^. It is also known that miRNA plays an important role in the occurrence and development of cancer. For instance, hsa-mir-204 was shown to be associated with several types of cancer, including melanoma^48^, breast cancer^49^ and liver cancer^50^. It was also shown that hsa-mir-128-3p can increase the sensitivity of colorectal cancer cells to chemotherapy^51^. In addition, hsa-mir-128-3p was significantly associated with resistance to chemotherapeutic agents. Nonetheless, there are few related reports on THCA. It is also noteworthy that hsa-mir-214 was reported to play a crucial role in regulating the proliferation and metastasis of papillary THCA cells^52^. Additionally, hsa-mir-150 and hsa-mir-338 were associated with the proliferation and invasion of various tumors, including colorectal cancer^53^, non-small cell carcinoma^54^ and cervical cancer^55^. In THCA, these hub miRNAs as well as the related lncRNA and mRNA jointly affected the tumor microenvironment. Their effect may be closely related to immune cell infiltration and tumor invasion.

In this study, seven differentially expressed mRNAs (BCL2, KCTD15, CDH2, GPAM, ITPR1, TUB and RGS6) were identified. These genes play an important role in the TME of many cancers and affect the efficacy of immune responses towards tumors. For example, the antiapoptotic B-cell lymphoma 2 (BCL2) gene was shown to be a key player in the development and progression of various types of cancer, including pancreatic^56^ and prostate cancer^57^. The gene is also actively involved in many pathways. On the other hand, KCTD15 is a member of the emerging class of KCTD ((K) potassium Channel Tetramerization Domain containing) proteins. In addition, downregulation of KCTD15 was reported to induce apoptosis and cell death, suggesting that it has a role in cellular homeostasis and proliferation^58^. The regulatory relationship of mir-204 and Cadherin 2 (CDH2) has successfully established a ceRNA network in breast cancer^59^. Moreover, Glycerol-3-phosphate acyltransferase (GPAM) is a key enzyme in the biosynthesis of triacylglycerols and phospholipids. Furthermore, MSC-AS1 facilitates the progression of LUAD by sponging miR-33b-5p to up-regulate GPAM^60^. Previous studies also showed that BCL2 Apoptosis Regulator (BCL2)^61^ and Cadherin 2 (CDH2)^62^ play an important role in the pathogenesis of thyroid cancer. Therefore, the present study further combined these mRNAs with tumor-related lncRNAs and miRNAs in thyroid cancer, providing a basis for studying the common mechanisms of multiple genes in thyroid cancer.

In recent years, immunotherapy has been considered an exciting therapeutic strategy for various types of cancers^63^. PD-L1 engages PD-1 receptor and induces PD-1 signaling, which can promote the initiation of T cell-mediated immunosuppressive programs. In this study, our survival curve shows that PD1 may be related to a better prognosis trend. Some other cancer studies have shown that PD-L1 overexpression was related to favorable prognosis^64–66^. The regulatory relationship between PD1/PDL1 and immune cells plays a very meaningful role in the immune infiltration mechanism of tumors, but there is very little research in thyroid cancer. In this study, we further elaborated the correlation between PD1/PDL1 and a variety of immune cells in thyroid cancer. First, we found that PD1/PDL1 is significantly positively correlated with Activated CD4 T cells, Activated CD8 T cells and NK cells. This result is consistent with previous studies. Activated CD8 T cell has been verified to be a favorable prognostic factor for a variety of cancers^67–69^. In thyroid cancer, the tumor microenvironment infiltration of NK cells may regulate the expression of PD1/PDL1, which in turn affects the prognosis of patients^70^. Activated dendritic cells are central regulators of the adaptive immune response, and as such are necessary for T-cell-mediated cancer immunity. In recent years, immunotherapy resistance has become more and more common in tumors. Some pathways can increase the risk of recurrence of immunotherapy. For example, the loss of PTEN is associated with increased levels of CCL2 and VEGF, decreased T cell infiltration, and resistance to PD-1 blockade^71^. Alterations in β-catenin/WNT signaling caused decreased CCL4 production, which led to diminished infiltration of CD103 + dendritic cells and impaired anti-tumor immune responses^72^.

While the present study provided some insightful findings, it had a major limitation. Given that the study was based of Bioinformatics analyses, it lacked validation from in vivo and in vitro experiments. Nonetheless, the study identified five hubnets related to THCA and these may be associated with immune infiltration in cancer and tumor invasion. These results therefore provide more information on tumor invasion and mechanism of action in patients with THCA. The findings also highlight the possible targets for the treatment of THCA.

## Conclusions

The study identified a hubnet consisting of seven mRNAs (BCL2, KCTD15, CDH2, GPAM, ITPR1, TUB and RGS6) and five miRNAs, namely; hsa-mir-204, hsa-mir-128, hsa-mir-214, hsa-mir-150 and hsa-mir-338. Moreover, the ceRNA network was used to determine the relationship between miRNA, lncRNA and immune-related mRNA. Understanding of the molecular role of THCA in the tumor microenvironment is important in designing appropriate treatment options.

## Supplementary Information

Supplementary Table 1.

Supplementary Figures.

## Data Availability

The data used in this study is available in the TCGA database.

## Abbreviations

TCGA: The cancer genome atlas
UCSC: University of California Santa Cruz
KEGG: Kyoto encyclopedia of genes and genomes
GO: Gene ontology
DEGs: Differentially expressed genes

## References

[1] Siegel RL, Miller KD, Jemal A (2019). Cancer statistics, 2019. CA Cancer J. Clin..

[2] Wang Y, Huang H, Hu F, Li J, Zhang L, Pang H (2019). CITED1 contributes to the progression of papillary thyroid carcinoma via the Wnt/beta-catenin signaling pathway. Onco Targets Ther..

[3] Balkwill F, Mantovani A (2001). Inflammation and cancer: back to Virchow?. Lancet.

[4] Dunn GP, Bruce AT, Ikeda H, Old LJ, Schreiber RD (2002). Cancer immunoediting: from immunosurveillance to tumor escape. Nat. Immunol..

[5] Galluzzi L, Senovilla L, Zitvogel L, Kroemer G (2012). The secret ally: immunostimulation by anticancer drugs. Nat. Rev. Drug Discov..

[6] Ayers M, Lunceford J, Nebozhyn M, Murphy E, Loboda A, Kaufman DR (2017). IFN-gamma-related mRNA profile predicts clinical response to PD-1 blockade. J. Clin. Invest..

[7] Martinez-Lostao L, Anel A, Pardo J (2015). How do cytotoxic lymphocytes kill cancer cells?. Clin. Cancer Res..

[8] Kather JN, Suarez-Carmona M, Charoentong P, Weis CA, Hirsch D, Bankhead P, et al. Topography of cancer-associated immune cells in human solid tumors. *Elife* 2018; 7. 10.7554/eLife.36967 PMC6133554 30179157

[9] Mauri C, Menon M (2017). Human regulatory B cells in health and disease: therapeutic potential. J. Clin. Invest..

[10] Lindau D, Gielen P, Kroesen M, Wesseling P, Adema GJ (2013). The immunosuppressive tumour network: myeloid-derived suppressor cells, regulatory T cells and natural killer T cells. Immunology.

[11] Jia D, Li S, Li D, Xue H, Yang D, Liu Y (2018). Mining TCGA database for genes of prognostic value in glioblastoma microenvironment. Aging (Albany NY).

[12] Qu S, Yang X, Li X, Wang J, Gao Y, Shang R (2015). Circular RNA: A new star of noncoding RNAs. Cancer Lett..

[13] Newman AM, Liu CL, Green MR, Gentles AJ, Feng W, Xu Y (2015). Robust enumeration of cell subsets from tissue expression profiles. Nat. Methods.

[14] Barbie DA, Tamayo P, Boehm JS, Kim SY, Moody SE, Dunn IF (2009). Systematic RNA interference reveals that oncogenic KRAS-driven cancers require TBK1. Nature.

[15] Becht E, Giraldo NA, Lacroix L, Buttard B, Elarouci N, Petitprez F (2016). Estimating the population abundance of tissue-infiltrating immune and stromal cell populations using gene expression. Genome Biol..

[16] Aran D, Hu Z, Butte AJ (2017). xCell: Digitally portraying the tissue cellular heterogeneity landscape. Genome Biol..

[17] Meurette O, Mehlen P (2018). Notch Signaling in the Tumor Microenvironment. Cancer Cell.

[18] Kim J, Bae JS (2016). Tumor-associated macrophages and neutrophils in tumor microenvironment. Mediators Inflamm..

[19] Gajewski TF (2015). The next hurdle in cancer immunotherapy: Overcoming the non-t-cell-inflamed tumor microenvironment. Semin. Oncol..

[20] Sharma P, Allison JP (2015). The future of immune checkpoint therapy. Science.

[21] Fridman WH, Pages F, Sautes-Fridman C, Galon J (2012). The immune contexture in human tumours: Impact on clinical outcome. Nat. Rev. Cancer.

[22] Ritchie ME, Phipson B, Wu D, Hu Y, Law CW, Shi W (2015). limma powers differential expression analyses for RNA-sequencing and microarray studies. Nucl. Acids Res..

[23] Kanehisa M, Goto S (2000). KEGG: kyoto encyclopedia of genes and genomes. Nucl. Acids Res..

[24] Kanehisa M (2019). Toward understanding the origin and evolution of cellular organisms. Protein Sci..

[25] Kanehisa M, Furumichi M, Sato Y, Ishiguro-Watanabe M, Tanabe M (2021). KEGG: Integrating viruses and cellular organisms. Nucl. Acids Res..

[26] Gong J, Jiang H, Shu C, Hu MQ, Huang Y, Liu Q (2019). Integrated analysis of circular RNA-associated ceRNA network in cervical cancer: Observational study. Medicine (Baltimore).

[27] Kohl M, Wiese S, Warscheid B (2011). Cytoscape: Software for visualization and analysis of biological networks. Methods Mol. Biol..

[28] Rosvall M, Bergstrom CT (2010). Mapping change in large networks. PLoS ONE.

[29] Lundstrom K (2011). Micro-RNA in disease and gene therapy. Curr. Drug Discov. Technol..

[30] Ferre F, Colantoni A, Helmer-Citterich M (2016). Revealing protein-lncRNA interaction. Brief Bioinform..

[31] Wong N, Wang X (2015). miRDB: An online resource for microRNA target prediction and functional annotations. Nucl. Acids Res..

[32] Chou CH, Shrestha S, Yang CD, Chang NW, Lin YL, Liao KW (2018). miRTarBase update 2018: A resource for experimentally validated microRNA-target interactions. Nucl. Acids Res..

[33] Agarwal V, Bell GW, Nam JW, Bartel DP. Predicting effective microRNA target sites in mammalian mRNAs. *Elife* 2015; 4. 10.7554/eLife.05005 PMC4532895 26267216

[34] Lee J, Lee SG, Kim K, Yim SH, Ryu H, Lee CR (2019). Clinical value of lymph node ratio integration with the 8(th) edition of the UICC TNM classification and 2015 ATA risk stratification systems for recurrence prediction in papillary thyroid cancer. Sci. Rep..

[35] Perrier ND, Brierley JD, Tuttle RM (2018). Differentiated and anaplastic thyroid carcinoma: Major changes in the American Joint Committee on Cancer eighth edition cancer staging manual. CA Cancer J. Clin..

[36] Yu G, Wang LG, Han Y, He QY (2012). clusterProfiler: An R package for comparing biological themes among gene clusters. OMICS.

[37] Medina-Echeverz J, Hinterberger M, Testori M, Geiger M, Giessel R, Bathke B (2019). Synergistic cancer immunotherapy combines MVA-CD40L induced innate and adaptive immunity with tumor targeting antibodies. Nat. Commun..

[38] Li HM, Yang H, Wen DY, Luo YH, Liang CY, Pan DH (2017). Overexpression of LncRNA HOTAIR is associated with poor prognosis in thyroid carcinoma: A study based on TCGA and GEO data. Horm. Metab. Res..

[39] Wang Y, He H, Li W, Phay J, Shen R, Yu L (2017). MYH9 binds to lncRNA gene PTCSC2 and regulates FOXE1 in the 9q22 thyroid cancer risk locus. Proc. Natl. Acad. Sci. USA.

[40] Zhu H, Lv Z, An C, Shi M, Pan W, Zhou L (2016). Onco-lncRNA HOTAIR and its functional genetic variants in papillary thyroid carcinoma. Sci. Rep..

[41] Hanahan D, Weinberg RA (2011). Hallmarks of cancer: The next generation. Cell.

[42] Muers M (2011). RNA: Genome-wide views of long non-coding RNAs. Nat. Rev. Genet..

[43] Caley DP, Pink RC, Trujillano D, Carter DR (2010). Long noncoding RNAs, chromatin, and development. ScientificWorldJournal.

[44] Augoff K, McCue B, Plow EF, Sossey-Alaoui K (2012). miR-31 and its host gene lncRNA LOC554202 are regulated by promoter hypermethylation in triple-negative breast cancer. Mol. Cancer.

[45] Salmena L, Poliseno L, Tay Y, Kats L, Pandolfi PP (2011). A ceRNA hypothesis: The Rosetta Stone of a hidden RNA language?. Cell.

[46] Galon J, Bruni D (2019). Approaches to treat immune hot, altered and cold tumours with combination immunotherapies. Nat. Rev. Drug Discov..

[47] Patel N, Garikapati KR, Makani VKK, Nair AD, Vangara N, Bhadra U (2018). Regulating BMI1 expression via miRNAs promote Mesenchymal to Epithelial Transition (MET) and sensitizes breast cancer cell to chemotherapeutic drug. PLoS ONE.

[48] Diaz-Martinez M, Benito-Jardon L, Alonso L, Koetz-Ploch L, Hernando E, Teixido J (2018). miR-204-5p and miR-211-5p contribute to BRAF inhibitor resistance in melanoma. Cancer Res..

[49] Muller V, Oliveira-Ferrer L, Steinbach B, Pantel K, Schwarzenbach H (2019). Interplay of lncRNA H19/miR-675 and lncRNA NEAT1/miR-204 in breast cancer. Mol. Oncol..

[50] Yu Y, Wang Y, Xiao X, Cheng W, Hu L, Yao W (2019). MiR-204 inhibits hepatocellular cancer drug resistance and metastasis through targeting NUAK1. Biochem. Cell Biol..

[51] Liu T, Zhang X, Du L, Wang Y, Liu X, Tian H (2019). Exosome-transmitted miR-128-3p increase chemosensitivity of oxaliplatin-resistant colorectal cancer. Mol. Cancer.

[52] Liu F, Lou K, Zhao X, Zhang J, Chen W, Qian Y (2018). miR-214 regulates papillary thyroid carcinoma cell proliferation and metastasis by targeting PSMD10. Int. J. Mol. Med..

[53] Fan H, Liu X, Zheng WW, Zhuang ZH, Wang CD (2017). MiR-150 alleviates EMT and cell invasion of colorectal cancer through targeting Gli1. Eur. Rev. Med. Pharmacol. Sci..

[54] Lu W, Zhang H, Niu Y, Wu Y, Sun W, Li H (2017). Long non-coding RNA linc00673 regulated non-small cell lung cancer proliferation, migration, invasion and epithelial mesenchymal transition by sponging miR-150-5p. Mol. Cancer.

[55] Luan X, Wang Y (2018). LncRNA XLOC_006390 facilitates cervical cancer tumorigenesis and metastasis as a ceRNA against miR-331-3p and miR-338-3p. J. Gynecol. Oncol..

[56] Wang L, Jia Z, Xie D, Zhao T, Tan Z, Zhang S (2020). Methylation of HSP70 orchestrates its binding to and stabilization of BCL2 mRNA and renders pancreatic cancer cells resistant to therapeutics. Cancer Res..

[57] Renner W, Langsenlehner U, Krenn-Pilko S, Eder P, Langsenlehner T (2017). BCL2 genotypes and prostate cancer survival. Strahlenther Onkol..

[58] Smaldone G, Beneduce G, Incoronato M, Pane K, Franzese M, Coppola L (2019). KCTD15 is overexpressed in human childhood B-cell acute lymphoid leukemia. Sci. Rep..

[59] Wang X, Gao C, Feng F, Zhuang J, Liu L, Li H (2020). Construction and analysis of competing endogenous RNA networks for breast cancer based on TCGA dataset. Biomed. Res. Int..

[60] Li S, Yang S, Qiu C, Sun D (2021). LncRNA MSC-AS1 facilitates lung adenocarcinoma through sponging miR-33b-5p to upregulate GPAM. Biochem. Cell Biol..

[61] Kolenda T, Guglas K, Kopczynska M, Sobocinska J, Teresiak A, Blizniak R (2020). Good or not good: Role of miR-18a in cancer biology. Rep. Pract. Oncol. Radiother..

[62] Wan Y, Zhang X, Leng H, Yin W, Zeng W, Zhang C (2020). Identifying hub genes of papillary thyroid carcinoma in the TCGA and GEO database using bioinformatics analysis. PeerJ.

[63] Ribas A, Wolchok JD (2018). Cancer immunotherapy using checkpoint blockade. Science.

[64] Darb-Esfahani S, Kunze CA, Kulbe H, Sehouli J, Wienert S, Lindner J (2016). Prognostic impact of programmed cell death-1 (PD-1) and PD-ligand 1 (PD-L1) expression in cancer cells and tumor-infiltrating lymphocytes in ovarian high grade serous carcinoma. Oncotarget.

[65] Droeser RA, Hirt C, Viehl CT, Frey DM, Nebiker C, Huber X (2013). Clinical impact of programmed cell death ligand 1 expression in colorectal cancer. Eur. J. Cancer.

[66] Schalper KA, Velcheti V, Carvajal D, Wimberly H, Brown J, Pusztai L (2014). In situ tumor PD-L1 mRNA expression is associated with increased TILs and better outcome in breast carcinomas. Clin. Cancer Res..

[67] Schumacher K, Haensch W, Roefzaad C, Schlag PM (2001). Prognostic significance of activated CD8(+) T cell infiltrations within esophageal carcinomas. Cancer Res..

[68] Farhood B, Najafi M, Mortezaee K (2019). CD8(+) cytotoxic T lymphocytes in cancer immunotherapy: A review. J. Cell Physiol..

[69] Szabo PA, Levitin HM, Miron M, Snyder ME, Senda T, Yuan J (2019). Single-cell transcriptomics of human T cells reveals tissue and activation signatures in health and disease. Nat. Commun..

[70] Delivanis DA, Gustafson MP, Bornschlegl S, Merten MM, Kottschade L, Withers S (2017). Pembrolizumab-induced thyroiditis: Comprehensive clinical review and insights into underlying involved mechanisms. J. Clin. Endocrinol. Metab..

[71] Peng W, Chen JQ, Liu C, Malu S, Creasy C, Tetzlaff MT (2016). Loss of PTEN promotes resistance to t cell-mediated immunotherapy. Cancer Discov..

[72] Spranger S, Bao R, Gajewski TF (2015). Melanoma-intrinsic beta-catenin signalling prevents anti-tumour immunity. Nature.

