# Supplementary material for: Identification of prognostic biomarkers related to the tumor microenvironment in thyroid carcinoma
Source: Sci Rep. 2021 Aug 10;11:16239. doi: 10.1038/s41598-021-90538-3 (PMC8355328; doi:10.1038/s41598-021-90538-3)
Supplement: Supplementary file 1 — Supplementary Table 1. [file 41598_2021_90538_MOESM1_ESM.docx]

| MiRNA | The predicted transcription factors |
| --- | --- |
| hsa-mir-204 | [ZNF341](https://www.genecards.org/cgi-bin/carddisp.pl?gene=ZNF341)/[JUND](https://www.genecards.org/cgi-bin/carddisp.pl?gene=JUND)/[SCRT1](https://www.genecards.org/cgi-bin/carddisp.pl?gene=SCRT1)/[TRIM28](https://www.genecards.org/cgi-bin/carddisp.pl?gene=TRIM28)/[EZH2](https://www.genecards.org/cgi-bin/carddisp.pl?gene=EZH2) |
| hsa-mir-128 | [SP1](https://www.genecards.org/cgi-bin/carddisp.pl?gene=SP1)/[BCL11A](https://www.genecards.org/cgi-bin/carddisp.pl?gene=BCL11A)/[IRF4](https://www.genecards.org/cgi-bin/carddisp.pl?gene=IRF4)/[EBF1](https://www.genecards.org/cgi-bin/carddisp.pl?gene=EBF1)/[CBFB](https://www.genecards.org/cgi-bin/carddisp.pl?gene=CBFB) |
| hsa-mir-214 | [CEBPB](https://www.genecards.org/cgi-bin/carddisp.pl?gene=CEBPB)/[MAX](https://www.genecards.org/cgi-bin/carddisp.pl?gene=MAX)/[EP300](https://www.genecards.org/cgi-bin/carddisp.pl?gene=EP300)/[GABPA](https://www.genecards.org/cgi-bin/carddisp.pl?gene=GABPA)/[STAT3](https://www.genecards.org/cgi-bin/carddisp.pl?gene=STAT3) |
| hsa-mir-150 | [CHD2](https://www.genecards.org/cgi-bin/carddisp.pl?gene=CHD2)/[ZBTB10](https://www.genecards.org/cgi-bin/carddisp.pl?gene=ZBTB10)/[SP1](https://www.genecards.org/cgi-bin/carddisp.pl?gene=SP1)/[MXD4](https://www.genecards.org/cgi-bin/carddisp.pl?gene=MXD4)/[FEZF1](https://www.genecards.org/cgi-bin/carddisp.pl?gene=FEZF1) |
| hsa-mir-338 | [USF2](https://www.genecards.org/cgi-bin/carddisp.pl?gene=USF2)/[KLF1](https://www.genecards.org/cgi-bin/carddisp.pl?gene=KLF1)/[HIC1](https://www.genecards.org/cgi-bin/carddisp.pl?gene=HIC1) |

**Supplementary table 1. The predicted** **transcription factors that regulate miRNAs.**
