# Supplementary material for: Identification of prognostic biomarkers related to the tumor microenvironment in thyroid carcinoma
Source: Sci Rep. 2021 Aug 10;11:16239. doi: 10.1038/s41598-021-90538-3 (PMC8355328; doi:10.1038/s41598-021-90538-3)
Supplement: Supplementary file 2 — Supplementary Figures. [file 41598_2021_90538_MOESM2_ESM.docx]

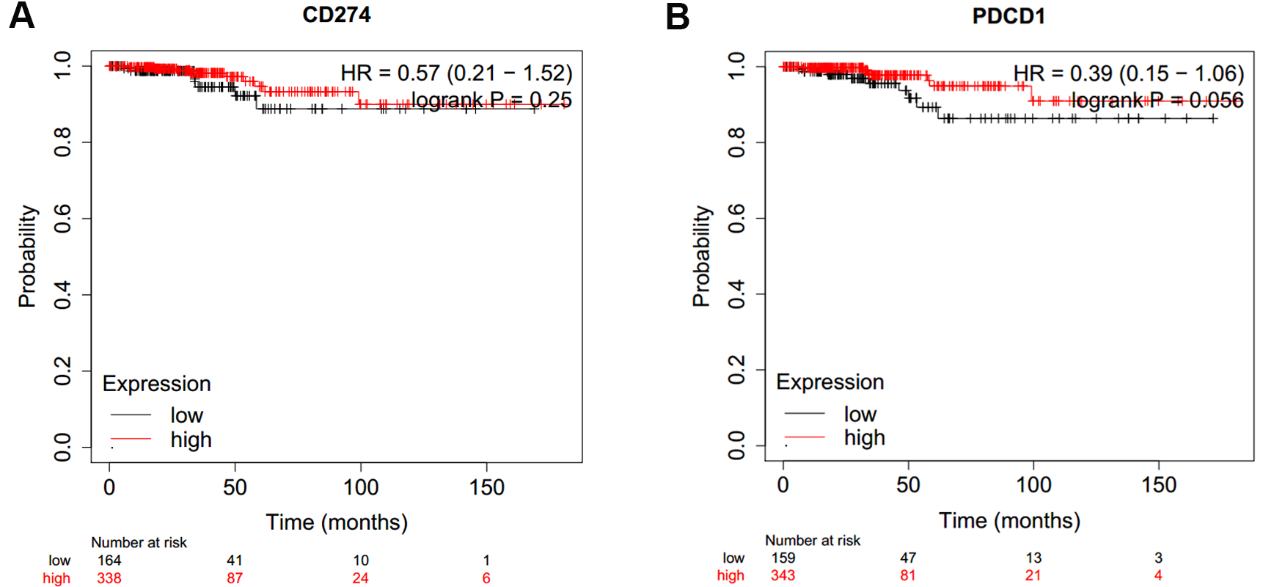


**Supplementary Figure 1: Survival analysis of PD-1/PD-L1.**


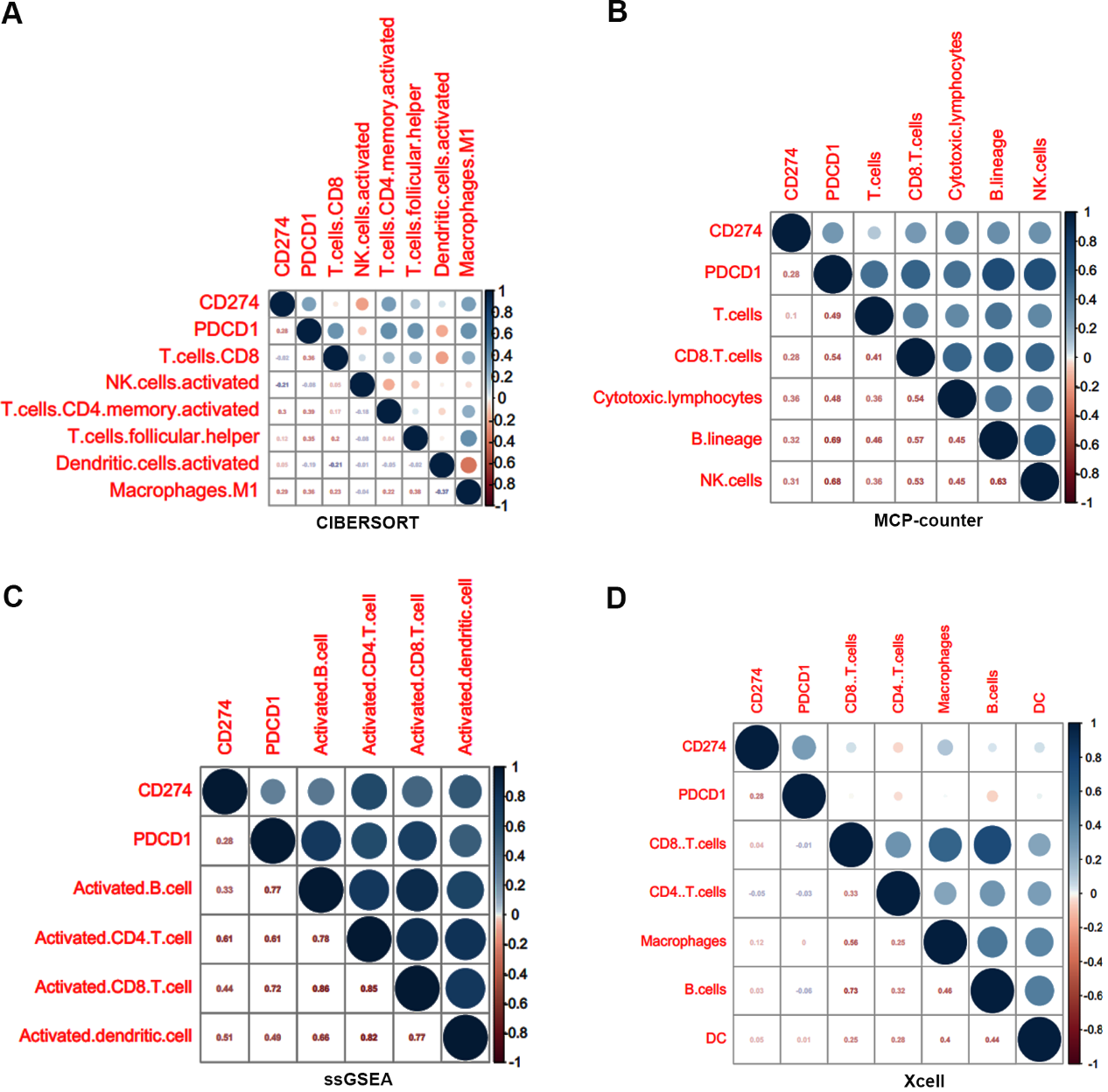


**Supplementary Figure 2: Correlation between PD-1/PD-L1 and immune cells.** The correlation between PD-1/PD-L1 and immune cells was assessed using four different methods and is shown in A-D. Blue represents positive correlation and red depicts negative correlation.


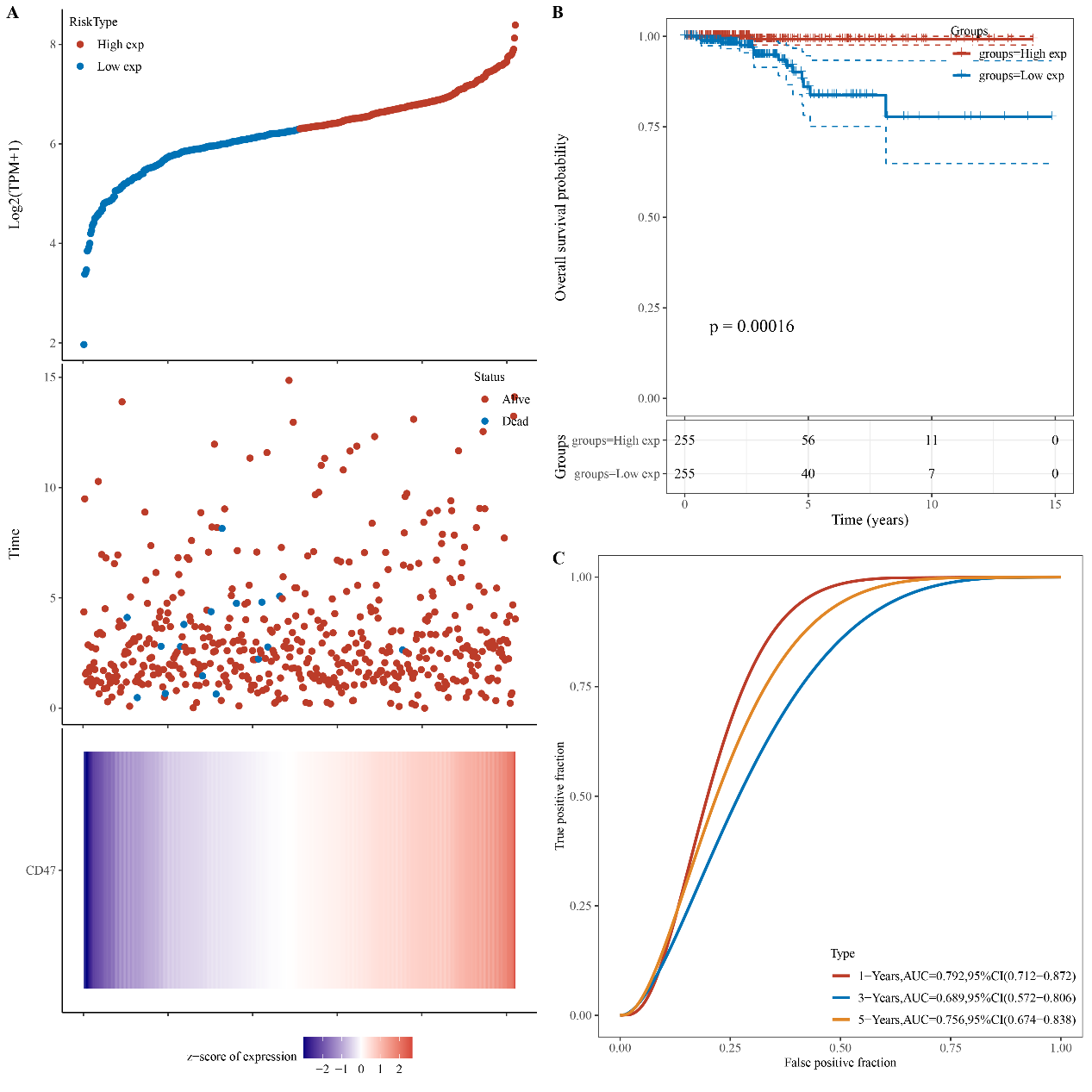


**Supplementary Figure 3: The relationship between CD47 and the prognosis of thyroid cancer.** (A) Take the median of the expression amount to divide the sample into high and low groups. (B&C) CD47 survival curve and ROC curve.


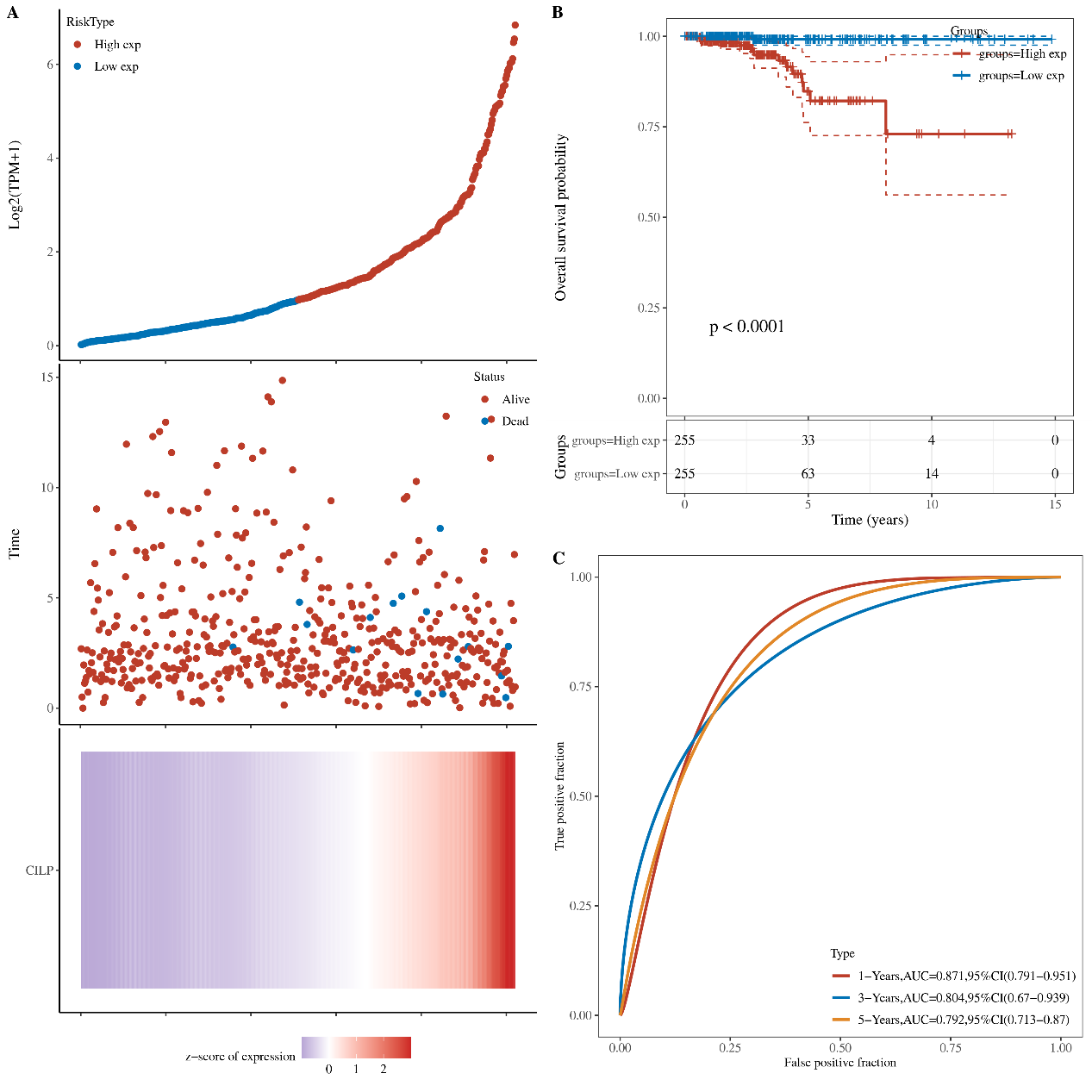


**Supplementary Figure 4: The relationship between CILP and the prognosis of thyroid cancer.** (A) Take the median of the expression amount to divide the sample into high and low groups. (B&C) CILP survival curve and ROC curve.


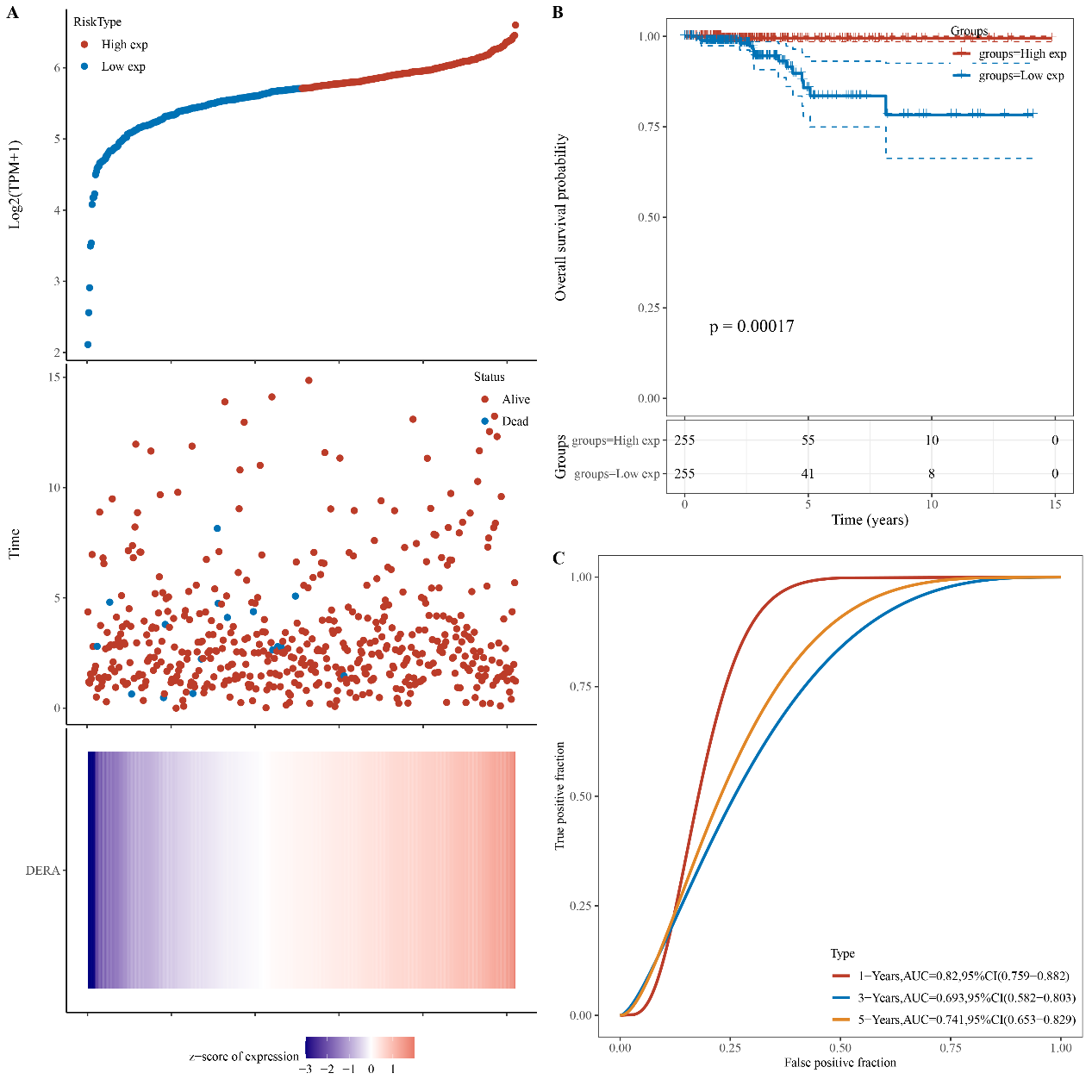


**Supplementary Figure 5: The relationship between DERA and the prognosis of thyroid cancer.** (A) Take the median of the expression amount to divide the sample into high and low groups. (B&C) DERA survival curve and ROC curve.


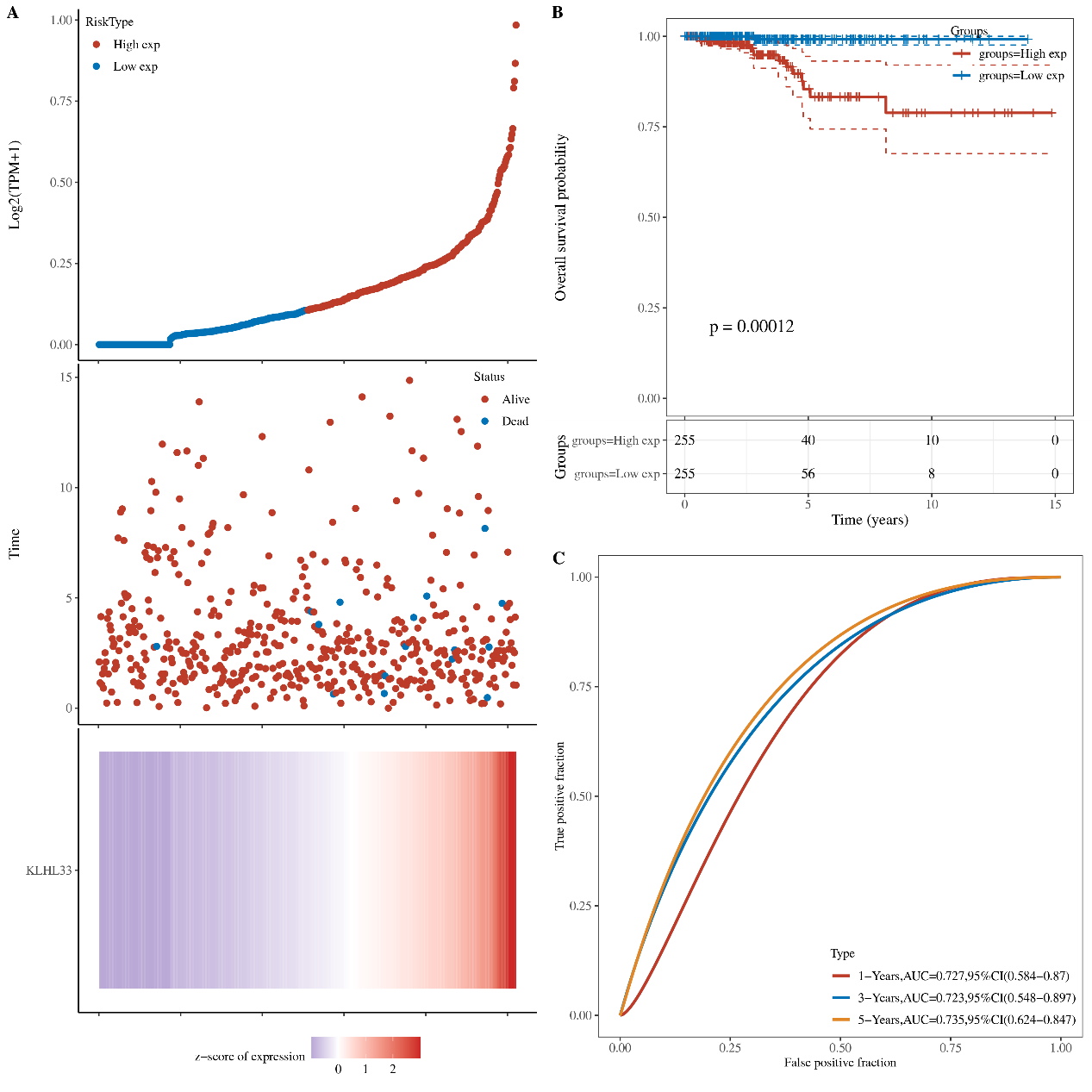


**Supplementary Figure 6: The relationship between KLHL33 and the prognosis of thyroid cancer.** (A) Take the median of the expression amount to divide the sample into high and low groups. (B&C) KLHL33 survival curve and ROC curve.


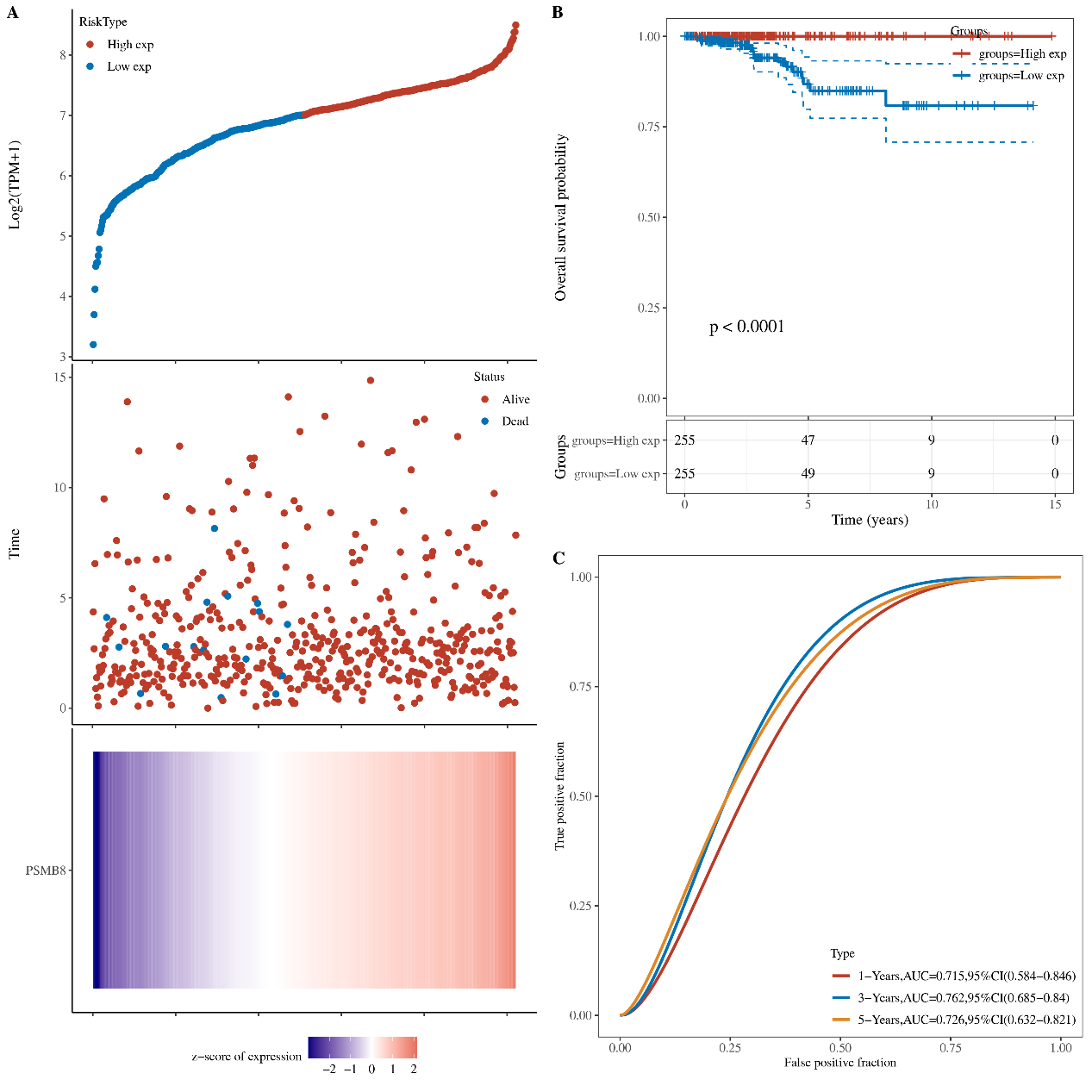


**Supplementary Figure 7: The relationship between PSMB8 and the prognosis of thyroid cancer.** (A) Take the median of the expression amount to divide the sample into high and low groups. (B&C) PSMB8 survival curve and ROC curve.
